# Local Structure and Surface Acidity of Overlayers Prepared by Atomic Layer Deposition of Silica on Alumina

**DOI:** 10.1021/acs.chemmater.5c01832

**Published:** 2025-09-30

**Authors:** Melis Yarar, Zixuan Chen, Diana Piankova, Matthias Becker, Alexander V. Yakimov, Christophe Copéret, Pierre Florian, Christoph R. Müller, Alexey Fedorov

**Affiliations:** † Department of Mechanical and Process Engineering, ETH Zürich, CH-8092 Zürich, Switzerland; ‡ Department of Chemistry and Applied Biosciences, ETH Zürich, CH-8093 Zürich, Switzerland; § CNRS, CEMHTI UPR3079, Université d’Orléans, 45071 Orléans, France

## Abstract

Understanding the structure of the silica–alumina interface and the reactivity of such interfacial sites in amorphous aluminosilicate (ASA) materials is essential due to their industrial utilization as solid acid catalysts. Here, we link the structure of silica layers grown on alumina by atomic layer deposition (ALD) to the acidic and catalytic properties of ASA. In particular, we study the local structure of silica overlayers as a function of the number of ALD cycles applied and reveal how the coordination environment of Al and Si sites governs the Lewis and Bro̷nsted acidity and catalytic activity using methanol dehydration as a model structure-sensitive reaction. Relying on advanced solid-state NMR characterization, including ^27^Al­{^29^Si} dipolar heteronuclear multiple-quantum coherence (D-HMQC) and ^29^Si­{^27^Al} dipolar-mediated refocused insensitive nuclei enhanced by polarization transfer (D-RINEPT) experiments, dynamic nuclear polarization surface-enhanced NMR spectroscopy (DNP SENS), and infrared spectroscopy using probe molecules (CO, pyridine), we demonstrate that the atomic-scale mixing of silica and alumina generates strong Bro̷nsted acidity and increases the strength of the Lewis acid sites. Our findings indicate that the density of acid sites is closely related to the coverage of the alumina surface by silica and can be controlled by the number of ALD cycles applied. This study advances our understanding of the relationship between the local environment of Si and Al sites, the abundance and strength of acid sites, and the superior high-temperature selectivity of SiO_*x*_-Al_2_O_3_-based catalysts in methanol dehydration when compared to unmodified alumina.

## Graphical abstract

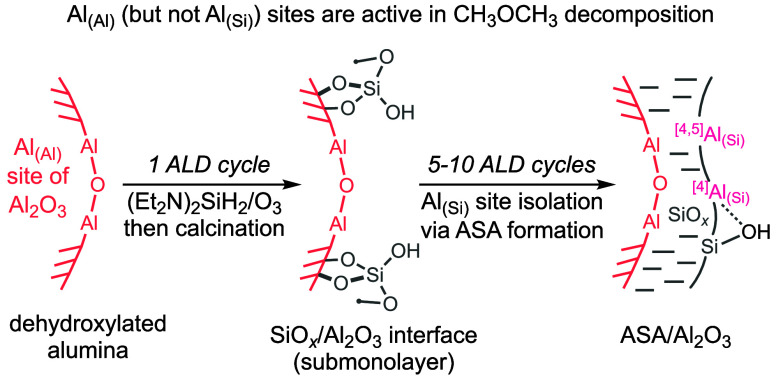

## Introduction

Amorphous silica–alumina (ASA) materials are widely utilized in heterogeneous catalysis owing to the tunable strength and abundance of their Bro̷nsted and Lewis acid sites (BAS and LAS, respectively).^1^ Combined with a high specific surface area, the acidic properties of ASAs foster their catalytic applications in petrochemistry, biomass conversions, and fine chemicals, to name just a few.^1,2^ Notably, the local coordination environment of Si and Al sites in ASA governs their surface acidity properties.^3,4^ For example, Lewis acidity in ASA is associated with Al^3+^ sites, which can be tri-, tetra-, and pentacoordinate (^[3]^Al^3+^, ^[4]^Al^3+^, and ^[5]^Al^3+^, respectively).^3,5–7^ Generally, the LAS strength depends on the coordination number of Al^3+^; viz., sites on dehydroxylated alumina with a lower coordination number provide stronger LAS.^8–10^ While ^[3]^Al_(Si)_^3+^ sites (such as (≡SiO)_3_Al) sites) have not been unequivocally characterized in ASA materials, such sites are expected to be strong LAS.^11,12^ ^[4]^Al^3+^ sites in ASA can be strong or medium LAS; in contrast, ^[5]^Al^3+^ sites have been associated with weaker LAS.^4,6,13,14^ Similarly, the emergence and strength of BAS in ASAs depend on the local coordination environment of the silanol (Si–OH) and Al^3+^ sites,^15^ which necessitates a proximity of the interacting sites, as found in pseudobridging Al^3+^···(μ^2^–OH)–Si silanols (strong BAS).^12,15–18^ The coordination geometry of Al sites in strong BAS has been proposed to be ^[4]^Al^3+^.^13^ Alternatively, pseudobridging silanols coordinated to a ^[5]^Al^3+^ site or two neighboring ^[4]^Al^3+^ and ^[5]^Al^3+^ sites interacting with the same silanol have also been suggested to yield strong BAS.^19–22^ Yet, the dependence of acid properties of ASA on the structure of the second coordination shell of the Al^3+^ sites, i.e., the relative ratio of Si and Al atoms and coordination number of Al atoms in ^[*n*]^Al^3+^_(Si/Al)_ (*n* = 4, 5, 6), is understood (and therefore controlled) to a lesser extent.^23,24^

Disentangling complex local structures, such as those in ASA, has been greatly aided by element-specific magic angle spinning nuclear magnetic resonance spectroscopy (MAS NMR).^25–28^ In addition to the insights discussed above, advanced two-dimensional NMR at an ultrahigh magnetic field strength (35.2 T) has revealed that pseudobridging silanols in ASA coexist with distinct strong BAS, similar to Si–OH–Al sites in zeolites.^28,29^ Dynamic nuclear polarization surface enhanced NMR spectroscopy (DNP SENS) provides additional structural information about surface sites due to sensitivity enhancement.^30,31^ For instance, results of a recent DNP SENS study suggested that a ^[4]^Al^3+^···(μ^2^–OH)–Si connectivity accounts for the main pseudobridging silanol structure.^25^ Surface acidity properties are typically assessed by NH_3_ temperature-programmed desorption experiments or Fourier transform infrared spectroscopy studies using basic probe molecules, such as pyridine (Py-FTIR).^27^ However, the complementary use of ^15^N-labeled pyridine (Py) allows the extraction of detailed acidity information from ^15^N DNP SENS spectra because this method is particularly sensitive to BAS.^32^ This sensitivity is attributed to the direct N–H bond in the pyridinium cation, which provides enhanced NMR signal intensity.^33^ In addition, ^15^N DNP SENS experiments are performed at a low temperature, allowing surface diffusion to be slowed down; for instance, ^15^N DNP SENS allowed the resolution of two strong BAS in AlO_*x*_/SiO_2_ and GaO_*x*_/SiO_2_ materials.^24,34^

Given that surface acidity is a key property for the catalytic application of ASAs, controlling the strength and relative distribution of LAS and BAS is essential yet challenging.^5,35,36^ In this context, atomic layer deposition (ALD) has emerged as a method of choice for tuning the surface properties of mixed oxides.^37–39^ Here, the tuning of the strength and distribution of LAS and BAS relies on understanding the atomic-scale interaction between the support and the ALD precursor, which can be enhanced by utilizing a dehydroxylated, supporting oxide to favor grafting reactions in preference over hydrolysis of the ALD precursors by the physisorbed water.^24,34^ The grafting reaction typically entails the interaction of alkyl or amide ligands of the ALD precursor with surface hydroxyls or oxo groups.^5,40^ The deposition of AlO_*x*_ overlayers is one of the most studied ALD reactions.^41^ In particular, we previously employed this approach to deposit trimethylaluminum on dehydroxylated silica, obtaining AlO_*x*_ overlayers after ozonolysis and calcination steps.^24^ The first ALD cycle yields mostly tetra- and pentacoordinate Al sites with 3–4 and 2 Si atoms in the second coordination sphere, respectively, i.e., ^[4]^Al_(3–4Si)_ and ^[5]^Al_(2Si)_ sites. The resulting AlO_*x*_-SiO_2_ material displayed strong Bro̷nsted acidity. However, consecutive ALD cycles led to the formation of amorphous alumina layers with a higher concentration of ^[5]^Al_(Al)_ sites. Such AlO_*x*_ overlayers showed a decreased relative amount of strong BAS and increased relative amounts of LAS of lower strength relative to the ASA interphase formed when applying 1 ALD cycle.^24^ Similar observations regarding the strength and abundance of acid sites with the number of applied ALD cycles were made for GaO_*x*_ overlayers on SiO_2_.^34^

Here, we engineer model surfaces and interfaces by applying a varying number of ALD cycles (1, 5, or 10) of bis­(diethylamino)­silane onto dehydroxylated γ-Al_2_O_3_. By controlling the number of ALD cycles, we obtained three materials referred to as Si1-, Si5-, and Si10–Al_2_O_3_ with surface sites that differ in the relative strength and abundance of LAS and BAS. The local structure of the Al sites was refined through MAS NMR experiments, particularly ^29^Si DNP SENS, ^27^Al­{^29^Si} dipolar-mediated heteronuclear multiple-quantum coherence (D-HMQC) and ^29^Si­{^27^Al} dipolar-mediated refocused insensitive nuclei enhanced by polarization transfer (D-RINEPT) with Carr–Purcell–Meiboom–Gill (CPMG) detection. These structural insights allowed us to rationalize the acidity properties of the prepared materials and their relevance for the catalytic dehydration of methanol to dimethyl ether. In stark contrast to the deposition of AlO_*x*_ on amorphous dehydroxylated silica, which features a decreasing BAS-to-LAS ratio with increasing ALD cycles,^24^ this ratio first increases for up to approximately 5 ALD cycles when SiO_*x*_ is deposited onto dehydroxylated alumina and only then decreases. Another notable difference is the increasing strength of LAS with the number of ALD cycles applied. Comparing Al_2_O_3_ to Si1-, Si5-, and Si10–Al_2_O_3_ in methanol dehydration reveals that, at temperatures ≤350 °C, unmodified Al_2_O_3_ and Si1–Al_2_O_3_ exhibit higher space-time yields of dimethyl ether (DME) than Si5-, and Si10–Al_2_O_3_. However, Si5- and Si10–Al_2_O_3_ appreciably outperform Si1–Al_2_O_3_ and Al_2_O_3_ at 450 °C due to a higher DME selectivity and lower selectivity to methane. Thus, the deposition of SiO_*x*_ onto Al_2_O_3_ allows for the chemical blocking of the unselective Al_(Al)_ sites of unmodified Al_2_O_3_.

## Experimental Section

### Materials and Methods

The three Si–Al_2_O_3_ materials studied in this work were synthesized by ALD at 300 °C using bis­(diethylamino)­silane (BDEAS) as the ALD precursor, alumina (Puralox, SBa 200, Sasol) dehydroxylated at 600 °C (denoted Al_2_O_3–600_) as the support, and ozone as the oxidant between subsequent ALD cycles; see the Supporting Information file for further details. The number of ALD cycles performed was 1, 5, or 10, and the as-deposited materials are denoted BDEAS1-, BDEAS5-, and BDEAS10-Al_2_O_3–600_, respectively. Treating the as-deposited materials at 600 °C for 3 h under a flow of synthetic air (50 mL min^–1^) yielded calcined materials denoted as Si1-, Si5-, and Si10–Al_2_O_3–600_. The calcined materials were handled without exposure to ambient air unless specified otherwise. The Supporting Information file contains details concerning the applied characterization methods, including inductively coupled plasma optical emission spectroscopy (ICP-OES), scanning/transmission electron microscopy (STEM and TEM), powder X-ray diffraction (XRD), N_2_ physisorption, ammonia temperature-programmed desorption (NH_3_-TPD), Py-FTIR, diffuse reflectance infrared Fourier transform spectroscopy with carbon monoxide used as the probe molecule (CO–DRIFTS), ^15^N and ^29^Si DNP SENS, and ^27^Al­{^29^Si} D-HMQC and ^29^Si­{^27^Al} D-RINEPT CPMG experiments. The catalytic activity in converting methanol to dimethyl ether was tested in a temperature range of 150–450 °C and at a space velocity of 2.90 h^–1^ using a Microactivity EFFI reactor (PID Eng&Tech). The progress of the reaction was probed by an online gas chromatograph (CompactGC 4.0, Gas Analyzer Solutions).

## Results

### Synthesis of Si–Al_2_O_3_

The materials Si1-, Si5-, and Si10–Al_2_O_3_ were prepared using 1, 5, or 10 ALD cycles, with BDEAS as the precursor and Puralox alumina dehydroxylated at 600 °C as the support; the deposition temperature was 300 °C (Figure 1a). The self-limiting surface saturation conditions for one ALD cycle were mapped by determining the number of BDEAS pulses required to consume all reactive aluminol (Al–OH) FTIR bands. For instance, while 5 pulses of BDEAS led to only a partial disappearance of the isolated aluminol bands at ca. 3795, 3774, 3729, and 3686 cm^–1^,^5^ 20 pulses of BDEAS resulted in a nearly complete disappearance of these bands (Figure S2). Hence, 20 pulses of BDEAS were considered sufficient to yield a surface saturation of the ALD precursor. The deposition of BDEAS is also evident from the appearance of characteristic CH_*x*_, SiH_*x*_, and Si­(NEt_2_)_*x*_ bands at ca. 3050–2770 cm^–1^, 2270 cm^–1^, and 1780–1315 cm^–1^, respectively (Figure 1b).^42^ Ozonolysis at 300 °C, applied after the BDEAS pulses, largely restored the OH intensity, while simultaneously decreasing the intensity of the CH_*x*_, SiH_*x*_, and Si­(NEt_2_)_*x*_ bands in BDEAS1-Al_2_O_3–600_ (Figure S2). A calcination treatment at 600 °C was performed to remove the remaining precursor surface species in BDEAS1-, BDEAS5-, and BDEAS10-Al_2_O_3–600_, yielding the materials Si1-, Si5-, and Si10–Al_2_O_3–600_ that do not feature any ALD precursor bands (Figure 1c). Consistent with the deposition of SiO_*x*_ onto Al_2_O_3–600_, FTIR spectra of Si5- and Si10–Al_2_O_3–600_ reveal a growing intensity of Si–O–Si vibrations at ca. 2150–1250 cm^–1^, which can, however, not be discerned in Si1–Al_2_O_3–600_. The intensity of the aluminol bands decreases with the number of ALD cycles applied, while in parallel, a band at 3743 cm^–1^ gradually appears, attributed to the formation of isolated silanols.^43^ In addition, the band at ca. 3595 cm^–1^ increases in intensity with the number of applied ALD cycles; this band is attributed to Si–OH interacting with LAS (Figure 1c).

**Figure 1:**
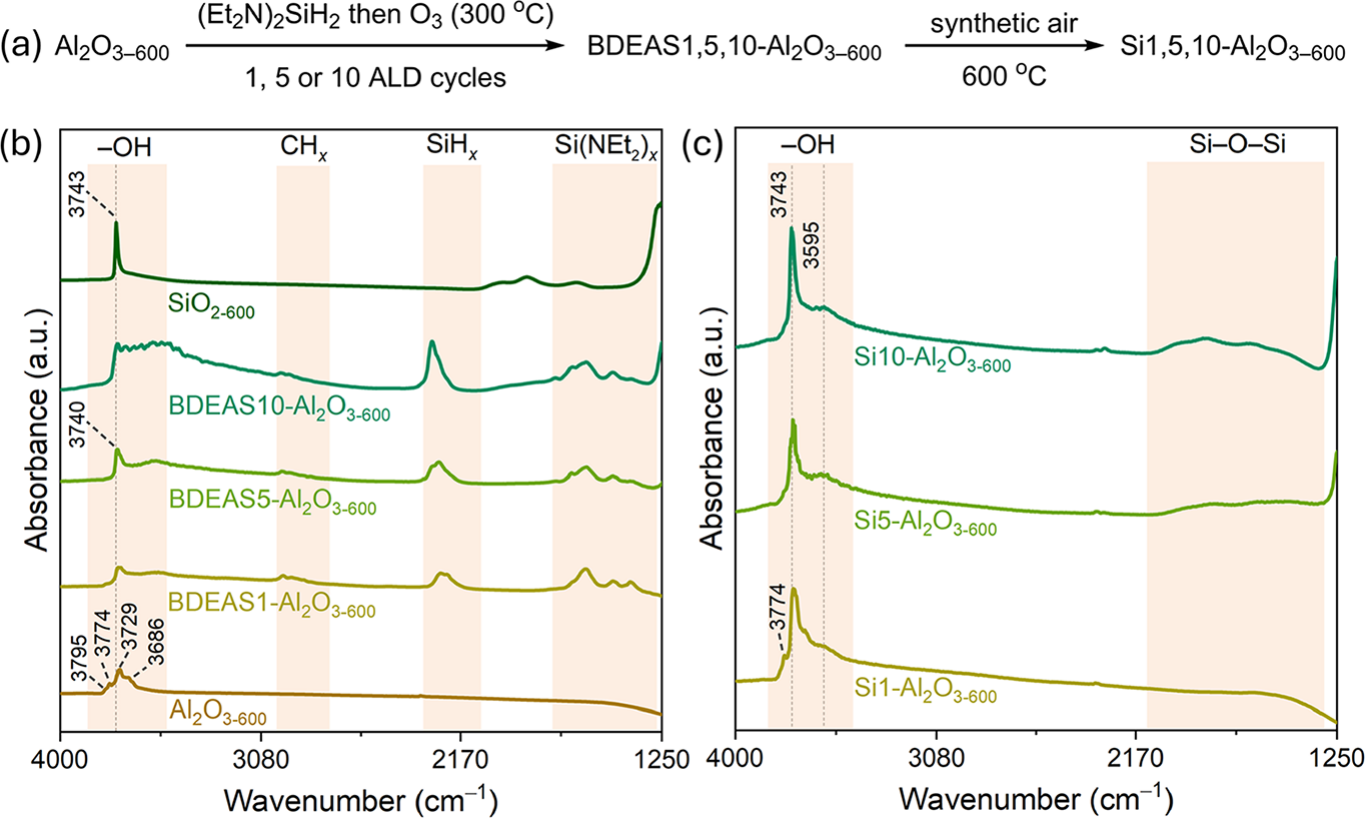
(a) Synthetic approach to obtain the Si–Al_2_O_3_ materials studied in this work; transmission FTIR spectra of (b) Al_2_O_3–600_, BDEAS1,5,10-Al_2_O_3–600_, and SiO_2–600_ and (c) Si1-, Si5, and Si10–Al_2_O_3–600_.

### Basic Characterization

To assess the Si content, the crystalline phase(s), surface area, and porosity of the prepared materials, ICP-OES, XRD, and N_2_ physisorption experiments were performed (after exposure to ambient air), respectively. ICP-OES determined that the Si weight fraction in Si1-, Si5-, and Si10–Al_2_O_3_ is 1.1, 5.9, and 9.9 wt %, respectively (Table S1). The surface area and pore volume evaluated by N_2_ physisorption and determined using the Brunauer–Emmett–Teller (BET) and Barrett–Joyner–Halenda (BJH) models (Figures S3 and S4) are presented in Table S1. The BET surface area increases with the number of ALD cycles from 150 m^2^ g^–1^ in Al_2_O_3–600_ to 154, 169, and 168 m^2^ g^–1^ for Si1-, Si5-, and Si10–Al_2_O_3–600_, which corresponds to 1.5, 8.3, and 13.7 Si nm^–2^, respectively (Table S1). The increase of the surface area with an increasing amount of SiO_*x*_ deposited onto alumina is likely due to surface amorphization induced by the ALD of SiO_*x*_ onto initially crystalline γ-Al_2_O_3_, as elaborated further below. Yet the pore volume of Al_2_O_3–600_ and Si1,5,10-Al_2_O_3–600_ was very similar, i.e., ca. 0.4 cm^3^ g^–1^. XRD patterns of Si1-, Si5-, and Si10–Al_2_O_3_, along with the references Al_2_O_3_ and SiO_2_, are given in Figure S5. The bulk structure of the alumina support, as probed by XRD, remained unchanged for all three prepared Si–Al_2_O_3_ materials (γ-Al_2_O_3_), i.e., no shifts in the peak positions were detected. Yet, for Si5- and Si10–Al_2_O_3–600_, a halo centered around a 2θ value of 21° that is attributed to amorphous SiO_2_ of the deposited silica layers was observed.

Further, using energy-dispersive X-ray spectroscopy (EDX) maps, we observed a homogeneous distribution of Si on the air-exposed Si1-, Si5-, and Si10–Al_2_O_3–600_ (Figure S6). To gain insight into the morphology of the deposited SiO_*x*_ layers at the nanoscale, we compared the initial material Al_2_O_3–600_ to Si10–Al_2_O_3–600_ using high-resolution transmission electron microscopy (HRTEM). Al_2_O_3–600_ contains particles with diameters of 1–2 μm, comprised of smaller, aggregated crystallites of approximately tens of nanometers in size (Figure 2a). However, Si10–Al_2_O_3–600_ features a significantly modified morphology relative to Al_2_O_3–600_ (Figure 2b). Specifically, despite the poor crystallinity of the crystallites in Al_2_O_3–600_, their surface is well-defined (Figure 2c). In contrast, on Si10–Al_2_O_3–600_, an amorphous surface layer of several nanometers in thickness is observed on the γ-Al_2_O_3_ crystallites (Figure 2b,d, overview in Figure S7), suggesting that the surface layer is very likely comprised of SiO_*x*_, i.e., there is no TEM-detectable surface exposure of the Al_2_O_3_ support. Such a core–shell microstructure is consistent with EDX line-scan analysis, which shows a higher Si intensity at (or close to) the surface (Figure S8).

**Figure 2:**
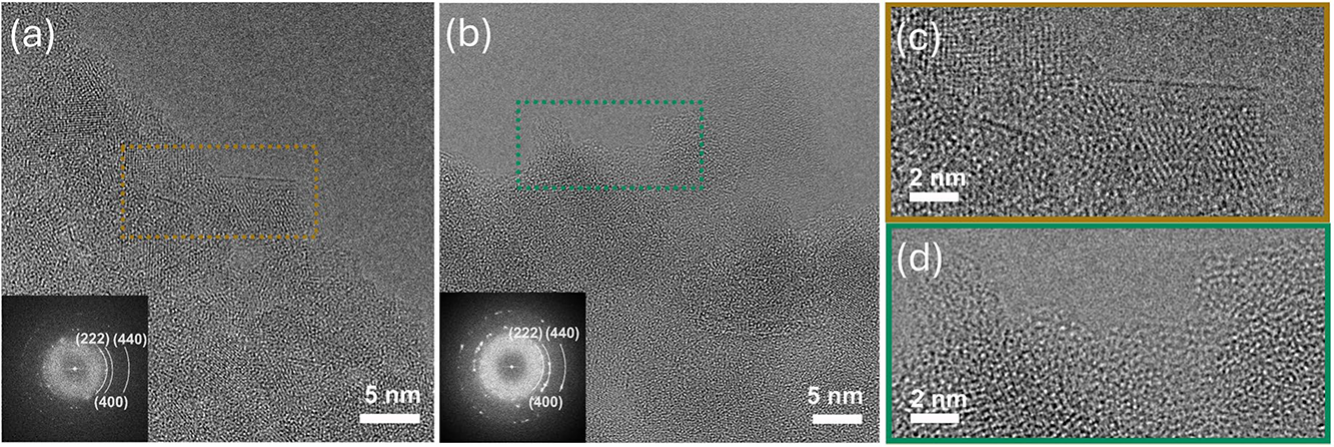
Overview HRTEM images of the surface of (a) Al_2_O_3–600_ and (b) Si10–Al_2_O_3–600_ with the corresponding FFT patterns indexed to γ-Al_2_O_3_ in insets. The panels (c, d) show HRTEM images magnified from (a) and (b), taken from the respective colored boxed areas.

### Methanol-to-DME Catalytic Tests

The catalytic performance of our Si–Al_2_O_3–600_ materials in the dehydration of methanol, i.e., methanol-to-dimethyl ether conversion, was evaluated by passing a flow of 0.75% CH_3_OH/N_2_ through a catalyst bed in the temperature range of 150–450 °C, with temperature increments of 100 °C. Using a weight hour space velocity (WHSV) of 2.90 h^–1^, the methanol conversion and product formation rate were monitored by GC for a duration of 70 min at each tested temperature. The catalytic results are summarized in Figure 3 and Table S3. At 150 °C, the methanol conversion was below 1.5% for all three Si–Al_2_O_3–600_ materials and the Al_2_O_3–600_ and SiO_2–600_ references. At 250 °C, the methanol conversion decreased with increasing Si loading, specifically from 76.4% for Al_2_O_3–600_ to 70.2, 37.3, and 25.1% for Si1-, Si5-, and Si10–Al_2_O_3–600_, respectively (Figure 3a). All catalysts exhibited a 100% selectivity toward DME (Table S3). Generally, the methanol conversion increased with temperature while the difference between the catalysts diminished such that at 450 °C, all of the Si–Al_2_O_3–600_ materials and Al_2_O_3–600_ displayed a methanol conversion close to the equilibrium conversion (ca. 80%),^44^ while SiO_2–600_ remained almost inactive.

**Figure 3:**
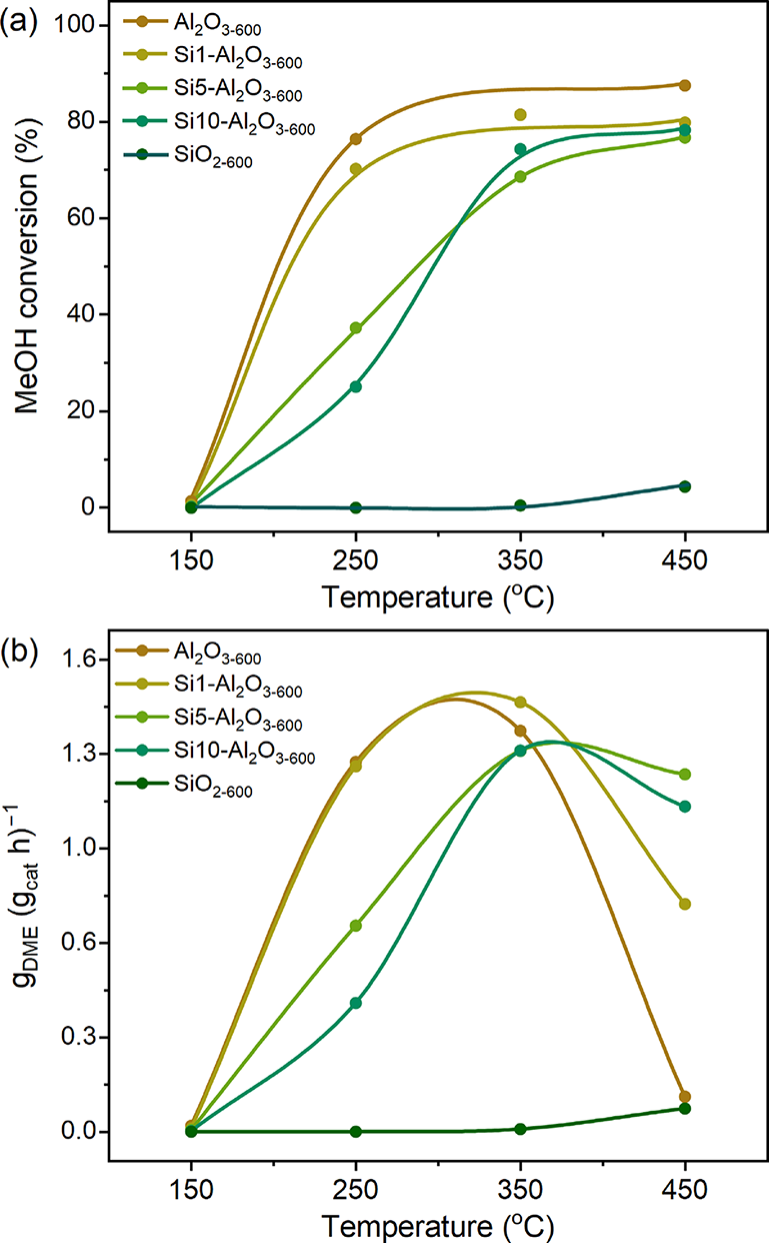
(a) Methanol conversion and (b) space-time yield of DME as a function of temperature under methanol dehydration conditions; the trendlines are added to guide the eye.

However, for all four catalysts, the space-time yield of DME (STY_DME_) at 450 °C is lower than at 350 °C (Figure 3b). The decrease in STY at 450 °C is most pronounced for Al_2_O_3–600_ and, to a lesser extent, for Si1–Al_2_O_3–600_, which show a STY_DME_ of 1.36 and 1.46 g_DME_ (g_cat_ h)^−1^ at 350 °C that decrease to 0.119 and 0.772 g_DME_ (g_cat_ h)^−1^ at 450 °C, respectively. For Si5- and Si10–Al_2_O_3–600_, the decrease in STY_DME_ is more modest, i.e., from 1.29 and 1.29 g_DME_ (g_cat_ h)^−1^ at 350 °C to 1.21 and 1.10 g_DME_ (g_cat_ h)^−1^ at 450 °C, respectively. The lower STY_DME_ at 450 °C parallels a decreasing selectivity for DME due to the formation of CH_4_ (Figure S9). In this context, the decomposition of DME to CH_4_, H_2_, and CO has been reported above 350 °C on Al_2_O_3_.^44,45^ Notably, we did not detect CO in our experiments, indicating that its concentration in the effluent gas is <500 ppm (the detection limit of CO by the GC used). Additionally, in all experiments, the carbon balance is in the range of 80–90%. All postreaction catalysts are gray, suggesting no appreciable coke deposition. Indeed, we did not observe Raman bands due to carbon in postreaction alumina (analyzed as a representative catalyst, Figure S16a). Attenuated total reflectance infrared spectrum of the postreaction alumina presented in Figure S16b displays an intense band at 1046 cm^–1^ and a weak band at 1578 cm^–1^, likely due to C–O and C=O vibrations, respectively, and no bands in the C–H regions. As the bands at 1046 and 1578 cm^–1^ are not observed on fresh alumina (exposed to air, Figure S16b), these are assigned to oxygenates such as carbonates or oxalates.^44–47^ Therefore, the low carbon balance is likely due to the formation of oxygenates that remain adsorbed on the surface of the catalysts at the explored reaction temperatures or cannot be quantified reliably by the employed GC method.^45^ Lastly, we note that while the catalytic tests discussed above were performed with catalysts loaded into reactors in air, a control experiment reveals similar catalytic performance for Al_2_O_3–600_ loaded in air and in pristine conditions (glovebox handling, see Table S3). As the adsorption energy of the reaction intermediates and products depends on the strength of the acid sites, in the following, we assess how the surface acidity changes with the thickness of the silica overlayer (controlled by the number of ALD cycles) and relate surface acidity to the local structure of the interacting Si and Al sites.

### Surface Acidity

Experiments using the basic probe molecules ammonia and pyridine were employed to investigate the surface acidity of the prepared materials.^48^ First, the total number of acid sites was quantified via NH_3_ TPD studies. Here, the air-exposed catalysts were pretreated in a flow of Ar at 600 °C, cooled down to 120 °C, and the dehydroxylated materials were exposed to a flow of 5% NH_3_/He for 30 min. NH_3_ desorption profiles were obtained during heating of the materials to 600 °C in a He flow (20 mL min^–1^) with a heating ramp of 10 °C min^–1^. The MS signal at *m*/*z* = 16 was used for quantification to prevent a possible contribution from water fragmentation.^49^ Figure 4a shows that on Al_2_O_3–600_, the peak in the NH_3_ desorption profile is centered around 250 °C. The desorption profiles for the Si–Al_2_O_3_ materials showed a broadening, and the intensity of the NH_3_ desorption peak gradually decreased with increasing Si content. There was no NH_3_ desorption from SiO_2–600_. This trend suggests a reduction in the total amount of surface acid sites with increasing Si loading, consistent with previous reports on Si deposition onto alumina.^14^ Quantifying the amount of NH_3_ desorbed, Table S4 demonstrates that 316 μmol of NH_3_ desorbed from one gram of Al_2_O_3–600_, corresponding to 1.26 NH_3_ molecules per nm^2^, which is comparable to the value reported previously for Puralox γ-Al_2_O_3_ pretreated at 550 °C (350 μmol g^–1^, 1.04 NH_3_ molecules per nm^2^).^50^ The amount of NH_3_ desorbed decreased to 259, 202, and 157 μmol g^–1^ for Si1-, Si5-, and Si10–Al_2_O_3–600_, respectively. The desorption profiles were deconvoluted using three components for Al_2_O_3–600_ and Si1–Al_2_O_3–600_ and two components for Si5–Al_2_O_3–600_ and Si10–Al_2_O_3–600_ (Figure S11). However, such a deconvolution does not allow the separation of the NH_3_ desorption peaks into LAS and BAS.

**Figure 4:**
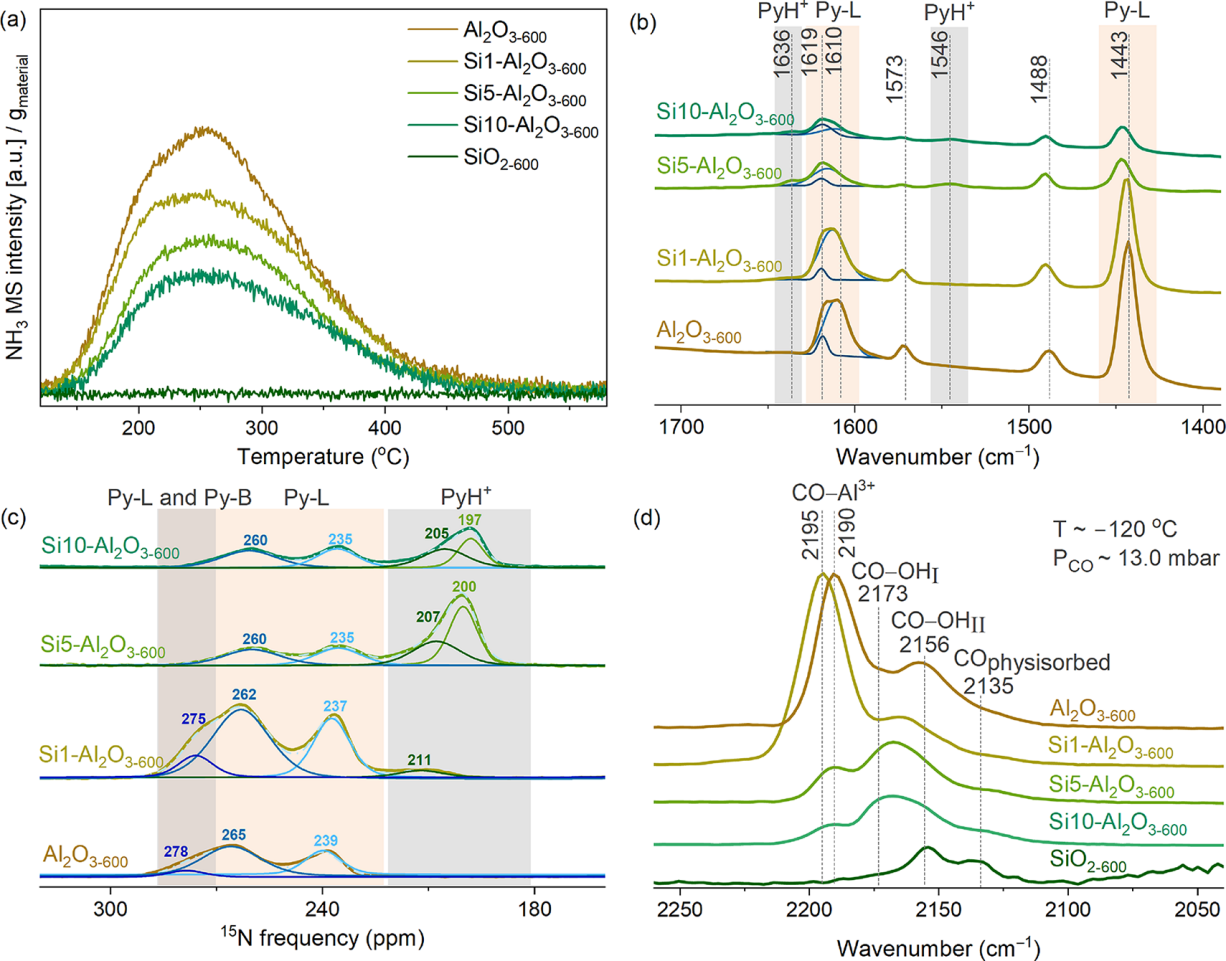
NH_3_ TPD profiles (a), Py-FTIR (b), ^15^N DNP SENS (c), and CO–DRIFTS (d) spectra of Al_2_O_3–600_, Si1-, Si5-, Si10–Al_2_O_3–600,_ and SiO_2–600_. In (b) and (c), ^15^N-Py was desorbed at 150 °C. PyH^+^, Py–L, and Py–B indicate protonated pyridine and pyridine interacting with LAS and weak BAS, respectively. The spectra in (d) were obtained under ca. 13 mbar of CO at *–*120 °C.

To allow for a discrimination of LAS and BAS, transmission FTIR and ^15^N DNP SENS experiments using pyridine as the probe molecule were used. Toward this end, dehydroxylated Al_2_O_3–600_, Si–Al_2_O_3–600_, and SiO_2–600_ were exposed to a vapor of ^15^N-Py at room temperature, followed by outgassing for 1 h at 150 °C and ca. 10^–5^ mbar. The bands due to Py adsorbed on LAS (Py-L) were observed in the regions 1460–1420 and 1630–1600 cm^–1^ (Figure 4b).^51^ Al_2_O_3–600_ features a broad band in the range 1630–1600 cm^–1^ that can be deconvoluted into two peaks centered at 1619 cm^–1^ and 1610 cm^–1^ (due to Py adsorbed on stronger and weaker LAS, minor and major contributions, respectively). With increasing Si loading, the contribution from the peak at 1610 cm^–1^ decreased gradually. For Si1–Al_2_O_3–600_, the band at 1610 cm^–1^ shifted to ca. 1615 cm^–1^, indicating that the deposition of a submonolayer SiO_*x*_ increased the strength of weak/medium alumina-based LAS. At the same time, the relative intensity of the band at 1619 cm^–1^ decreased in Si1–Al_2_O_3–600_ compared to Al_2_O_3–600_, consistent with the deposition of SiO_*x*_ onto strong LAS of Al_2_O_3–600_. For Si10–Al_2_O_3–600_, the contribution from the peak at 1619 cm^–1^ to the overall Py-L signal dominates. This observation suggests an increasing relative ratio of stronger to weaker LAS with increasing Si loading. Another clear trend is the decreasing intensity of the bands due to Py adsorbed on LAS (seen in both regions mentioned above) with increasing Si loading, a consequence of the covering of Al^3+^ sites by SiO_*x*_. No appreciable amount of Py was observed on SiO_2–600_ under the conditions employed (Figure S12). The bands of pyridinium (PyH^+^) at 1546 cm^–1^ and 1636 cm^–1^ were discerned for Si5- and Si10–Al_2_O_3–600_, indicating the emergence of strong Bro̷nsted acidity in these two materials; such bands were absent in Si1–Al_2_O_3–600_ and Al_2_O_3–600_.^51^ This trend is opposite to the one observed when layers of AlO_*x*_ were deposited onto SiO_2_ by ALD.^24^

The number of LAS in our materials can be estimated from the acquired Py-FTIR spectra utilizing the Beer–Lambert-Bouguer law using a reported molar absorption coefficient for Py on LAS (ε_Py–L_) of 1.87 cm μmol^–1^.^52,53^ For this analysis, we used unlabeled Py (see the SI file for details and Figure S12) and obtained 130 μmol g^–1^ of Py adsorbed on LAS in Al_2_O_3–600_, in agreement with the reported value of 125 μmol g^–1^ for γ-Al_2_O_3_ dehydroxylated at 450 °C.^53^ The amount of LAS on Si1–Al_2_O_3–600_ was similar to that of Al_2_O_3–600_, i.e. 135 μmol g^–1^ but decreased to 68 and 51 μmol g^–1^ for Si5- and Si10–Al_2_O_3–600_, respectively (Table S4). We limit the FTIR-based quantification to the Lewis acid sites as the extinction coefficient for PyH^+^ on ASA has not been reported, and the extinction coefficient for PyH^+^ on zeolites has been reported to depend on the zeolite structure, hindering an appropriate selection.^53^

The evolution of the strength of the LAS and BAS with increasing number of Si ALD layers was further refined through ^15^N DNP SENS experiments; the spectra and the peak fitting results are presented in Figure 4c and Table S5. The spectrum of Al_2_O_3–600_ shows peaks at ^15^N chemical shifts of 278, 265, and 239 ppm, consistent with previous observations for Al_2_O_3–500_.^54^ The peaks at 239 and 265 ppm are due to strong and medium strength LAS, respectively, while the minor peak at 278 ppm is due to Py adsorbed on either weak LAS or BAS.^54–58^ Si1–Al_2_O_3–600_ displayed more shielded signals, i.e., shifted to 275, 262, and 237 ppm. In addition to the peak shift, the relative intensity of the peak at 275 ppm increased, and a low-intensity peak due to PyH^+^ appeared at 211 ppm. The strength of the medium and strong LAS further increased in Si5- and Si10–Al_2_O_3–600_; viz., the peaks due to Py-L were further shifted to 260 and 235 ppm. The least shielded peak at 278–275 ppm disappeared for Si5- and Si10–Al_2_O_3–600_, while peaks due to two strong BAS appeared, i.e., two PyH^+^ peaks at 207 and 200 ppm for Si5- and at 205 and 197 ppm for Si10–Al_2_O_3–600_, respectively. Similarly, two strong BAS were reported for ALD-prepared Al-SiO_2–500_ and Ga-SiO_2–500_ materials.^24,34^

Lastly, CO–DRIFTS studies were utilized to refine changes in weak BAS and LAS with increasing Si content.^13^ In such experiments, the materials were dehydroxylated in a Harrick cell at 600 °C at ca. 10^–4^ mbar overnight; subsequently, CO was admitted to the cell at −120 °C in increments of 7 mbar until a CO partial pressure of ca. 100 mbar was achieved. The complete CO adsorption and desorption profiles are presented in Figures S13 and S14, while the profiles obtained at ca. 13 mbar CO pressure during CO adsorption are plotted in Figure 4d. Al_2_O_3–600_ featured an intense band at 2190 cm^–1^ corresponding to CO interacting with Al^3+^ sites.^59,60^ This band was blue-shifted to 2195 cm^–1^ in Si1–Al_2_O_3–600_ and to 2193 cm^–1^ in both Si5- and Si10–Al_2_O_3–600_. These wavenumbers are within the typical range of CO–Al^3+^ adducts on alumina (2190–2230 cm^–1^).^13,27,61^ It was reported that isolated Al^3+^ sites, such as corner or edge Al^3+^ sites of alumina or dispersed (isolated) Al^3+^ sites on ASA, form adducts at higher CO frequencies (ca. 2230–2215 cm^–1^) compared to bulk or higher coordinated Al^3+^ sites (2190–2196 cm^–1^).^61,62^ Based on these assignments, the observed shift to higher wavenumbers after Si deposition can be interpreted as an increase in the strength of LAS, which is associated with a decrease in the coordination number and isolation of Al^3+^ sites. With increasing number of ALD cycles, the intensity of the CO–Al^3+^ band reduced appreciably, in particular when comparing Al_2_O_3–600_ and Si1–Al_2_O_3–600_ to Si5- and Si10–Al_2_O_3–600_. The decreasing intensity of the CO–Al^3+^ band with increasing quantity of Si deposited is linked to the covering of Al^3+^ LAS with SiO_*x*_, consistent with the NH_3_ TPD, Py-FTIR, and ^15^N-Py-DNP SENS results discussed above. The presence of weak BAS on Al_2_O_3–600_, i.e., OH groups interacting with CO, was identified by a band centered at 2156 cm^–1^;^63^ this band was also observed on SiO_2–600_. On Si1-, Si5-, and Si10–Al_2_O_3–600_, a new band at, respectively, 2162, 2168, and 2173 cm^–1^ appeared. This band is ascribed to CO interacting with strong BAS.^63,64^ The shift to higher wavenumbers of this band indicates an increasing strength of BAS. These results confirm that the amount and strength of strong BAS increases with increasing number of Si ALD cycles and that the acidic strength of LAS on Al_2_O_3–600_ increases with Si deposition, while the amount of LAS decreases.

### Local Environment of Si

To assess the structure of Si atoms in Si1-, Si5-, and Si10–Al_2_O_3–600_, ^29^Si DNP SENS experiments using a contact time of 2 ms were performed. The resulting spectra and corresponding fittings are presented in Figure 5. The fitting results are compiled in Table S2. In general, three components were sufficient to fit all spectra. While the Si sites in Si5–Al_2_O_3–600_ and Si10–Al_2_O_3–600_ are qualitatively similar, the spectrum of Si1–Al_2_O_3–600_ is appreciably different. For Si1–Al_2_O_3–600_, the most intense peak is at a chemical shift of −82.6 ppm, while the less intense peaks are located at −89.1 and −92.8 ppm. Already for Si5–Al_2_O_3–600_, the peak at −85.9 ppm becomes a minor peak relative to the peaks centered at −92.4 and −99.9 ppm. The relative intensity of the peaks and their chemical shifts further evolve for Si10–Al_2_O_3–600_ such that the peak at −101.6 ppm is the most prominent, followed by the peak at −93.1 ppm and a low-intensity peak at −86.2 ppm. The more deshielded peak in Si1–Al_2_O_3–600_ is attributed to Si species with an increasing number of Al atoms in the second coordination sphere (such as Si_(3–4Al)_) and/or carrying OH groups.^65^ The higher degree of shielding for an increasing Si content (i.e., lower chemical shifts) suggests an increasing polymerization of the silica network incorporating fewer aluminum atoms.^66^

**Figure 5:**
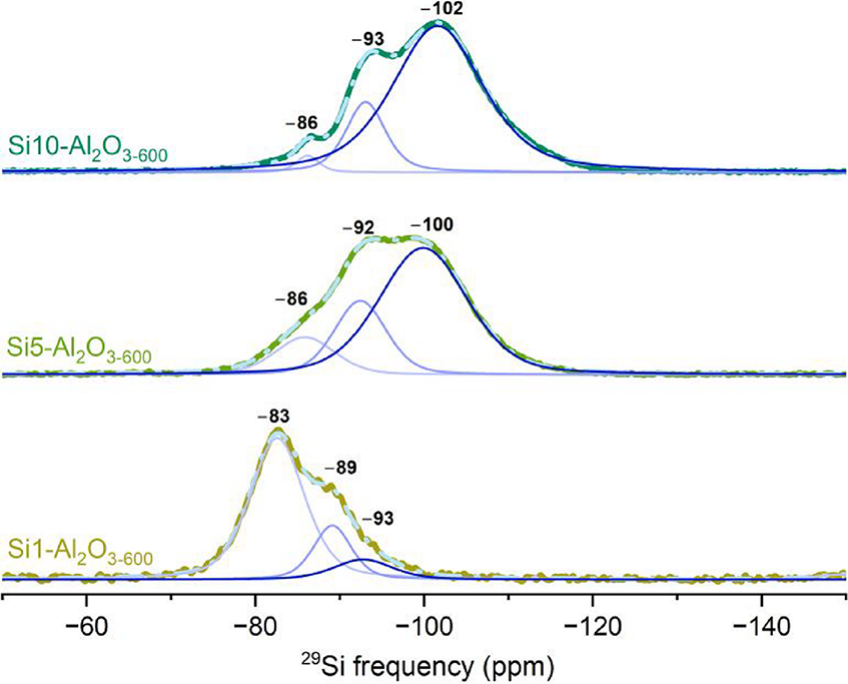
^29^Si DNP SENS data of Si1-, Si5-, and Si10–Al_2_O_3–600_. The simulation is presented as a dashed light-blue trace, with the individual fitted components shown as solid blue traces.

### Si–O–Al Connectivity

Figure 6a,b displays the spectra obtained for Si1- and Si10–Al_2_O_3–600_ using one-dimensional ^27^Al­{^29^Si} D-HMQC experiments at varying recoupling times, which allowed probing Al species at various distances from Si sites, since increasing the dipolar-based recoupling time extends the distances at which recoupling occurs.^67^ At a short recoupling time of 1.5 ms, these ^29^Si-filtered ^27^Al spectra mainly provide information about the SiO_*x*_/Al_2_O_3_ interface, i.e., Al atoms close to Si contribute primarily to the signal. Note that the sites of the SiO_*x*_/Al_2_O_3_ interface reside at the outer surface and subsurface for Si1- and Si10–Al_2_O_3–600_, respectively. Experiments with a prolonged recoupling time, e.g., 9.0 ms, probe the Al environments more distant from the Si atoms, i.e., further away from the SiO_*x*_/Al_2_O_3_ interface and peering deeper into Al_2_O_3_ and the ALD-grown SiO_*x*_ layer (vide infra). The presence of ^[4]^Al, ^[5]^Al, and ^[6]^Al sites, associated with peaks centered at approximately 60, 30, and 10 ppm, respectively, was observed for all tested recoupling times (1.5, 3.0, 6.0, and 9.0 ms) for both Si1- and Si10–Al_2_O_3–600_. However, in both Si1- and Si10–Al_2_O_3–600_, longer recoupling times showed an increasing contribution of ^[4]^Al and ^[5]^Al sites relative to the spectra acquired with a recoupling time of 1.5 ms. This increase is subtle for Si1–Al_2_O_3–600_, but more clearly observable for Si10–Al_2_O_3–600_. For Si1–Al_2_O_3–600_, this increase in the contribution of ^[4]^Al and ^[5]^Al sites can be due to probing more distant (from the Si at the interface) surface Al sites, consistent with a submonolayer Si coverage in this material. As ^[6]^Al_(Al)_ surface sites in γ-Al_2_O_3–600_ are OH-terminated, while ^[4]^Al_(Al)_ and ^[5]^Al_(Al)_ surface sites can either be OH-terminated or OH-free,^10,68^ we hypothesize that the grafting of BDEAS onto aluminols and subsequent calcination generate ^[4,5,6]^Al_(Al,Si)_ sites from ^[4,5,6]^Al_(Al)_–OH sites. At the same time, OH-free ^[4]^Al_(Al)_ and ^[5]^Al_(Al)_ remain mostly unmodified by ALD, and it is those sites that are probed at higher recoupling times. In contrast, for Si10–Al_2_O_3–600_, the observed increase in the contribution of ^[4]^Al and ^[5]^Al sites, as well as the increase in signal-to-noise ratio, can be explained by the diffusion of Al^3+^ into the deposited layer of SiO_*x*_, forming ASA. The relative intensity of ^[6]^Al sites decreases for a longer recoupling time in Si10–Al_2_O_3–600_ because such ^[6]^Al sites are located in the vicinity of Si sites, mainly at the SiO_*x*_/Al_2_O_3_ interface.

**Figure 6:**
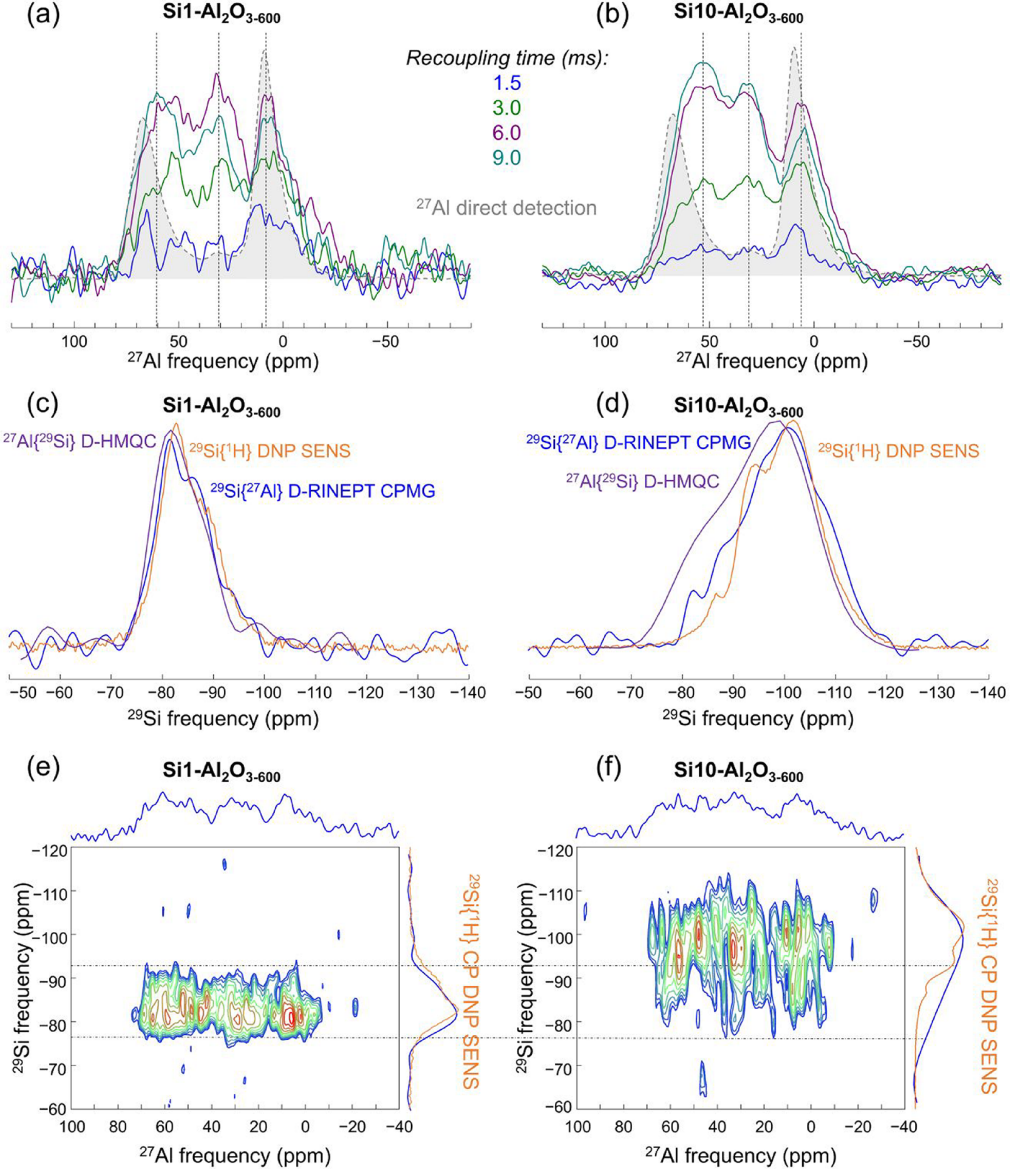
^27^Al­{^29^Si} SR4_1_^2^ D-HMQC spectra of (a) Si1–Al_2_O_3–600_ and (b) Si10–Al_2_O_3–600_ at 17.6 T using various recoupling times; for the 6.0 ms experiment, the S/N is 7.6 for Si1–Al_2_O_3–600_ and 10.6 for Si10–Al_2_O_3–600_. Direct detection ^27^Al spectrum of the Al_2_O_3_ reference is given in gray, and the dashed vertical lines are a guide for the eyes to indicate the position of the ^[4]^Al, ^[5]^Al, and ^[6]^Al sites, respectively. A comparison of ^29^Si­{^27^Al} SR4_1_^2^ D-RINEPT CPMG, ^29^Si­{^1^H} DNP SENS, and the ^29^Si projection of the 2D ^27^Al­{^29^Si} SR4_1_^2^ D-HMQC spectra (blue, orange, and purple, respectively) of (c) Si1–Al_2_O_3–600_ and (d) Si10–Al_2_O_3–600_. ^27^Al­{^29^Si} SR4_1_^2^ D-HMQC 2D correlation spectra of (e) Si1–Al_2_O_3–600_ and (f) Si10–Al_2_O_3–600_ obtained with a recoupling time of 6.0 ms. ^29^Si­{^1^H} DNP SENS spectra are shown in orange in the vertical projections. Horizontal dashed lines guide the eye to locate the maxima in the ^29^Si dimension.

The presence of ^[5]^Al sites and the broadening of all peaks differentiate the spectra of Si1- and Si10–Al_2_O_3–600_ from that of γ-Al_2_O_3–600_ (obtained via a direct detection measurement from the bulk of Si1- and Si10–Al_2_O_3–600_), indicating an appreciably larger distribution of local Al environments at or near the SiO_*x*_/Al_2_O_3_ interface in the Si-containing materials. Simulating experiments with a recoupling time of 6.0 ms provided a relative ratio of signal intensities around 69, 37, and 12 ppm, assigned to ^[4]^Al:^[5]^Al:^[6]^Al sites in Si1- and Si10–Al_2_O_3–600_, as 58:24:18 and 69:13:18, respectively (Figure S15). These results suggest that additional ALD cycles reorganize the (near-) surface structure of Si1–Al_2_O_3–600_ by increasing the amount of ^[4]^Al sites, which correlates with the increasing strength of LAS and BAS with Si loading. The low signal-to-noise (S/N) ratio for the experiments at short recoupling times makes the assessment of the local Al environments at the SiO_*x*_/Al_2_O_3_ interface challenging. However, the systematically lower S/N ratio for Si1–Al_2_O_3–600_ than for Si10–Al_2_O_3–600_ at a recoupling time of 1.5 ms suggests that a smaller total amount of Al atoms is located near Si in Si1–Al_2_O_3–600_; an observation that is consistent with a submonolayer coverage of the Al_2_O_3_ surface by SiO_*x*_ in Si1–Al_2_O_3–600_ and the diffusion of Al^3+^ into the SiO_*x*_ layer in Si10–Al_2_O_3–600_. The diffusion of Si atoms into crystalline Al_2_O_3_ particles is considered improbable as it is inconsistent with the absence of an appreciable decrease in the relative intensity of ^[5]^Al sites with increasing recoupling time for both Si1–Al_2_O_3–600_ and Si10–Al_2_O_3–600_. In contrast, the migration of Al^3+^ into the SiO_*x*_ layer increases the amount of Si in the vicinity of Al and therefore the signal increases.

It is worth noting that ^29^Si­{^1^H} DNP SENS mainly probes the outer surface of the deposited silica layer, whereas both the ^27^Al­{^29^Si} D-HMQC and ^29^Si­{^27^Al} D-RINEPT CPMG experiments are more selective to the SiO_*x*_/Al_2_O_3_ interface.^24,69^ The ^29^Si spectra of Si1–Al_2_O_3–600_ under various experimental conditions (^27^Al­{^29^Si} D-HMQC, ^29^Si­{^1^H} DNP SENS and ^29^Si­{^27^Al} D-RINEPT CPMG) are remarkably similar (Figure 6c). The similarity of the Si1–Al_2_O_3–600_ spectra obtained using these three methods indicates that one ALD cycle deposits a thin silica film in which all Si sites are in contact with the Al_2_O_3_ surface and the radical-containing impregnated DNP solution, which fills the particles’ intergrain space. This is in contrast with the result obtained for Si10–Al_2_O_3–600_, for which the ^29^Si­{^1^H} DNP SENS spectrum features higher relative intensity in the range between −100 ppm and ca. −110 ppm compared to the ^27^Al­{^29^Si} D-HMQC spectrum (Figure 6d). The peaks at −100 ppm and −110 ppm are associated with extended siloxane networks (Si–O–Si species) at the outer surface of the ALD-deposited layer, and mainly assigned to (AlO)_1_Si­(OSi)_3_ and Si­(OSi)_4_ sites, respectively.^70^ At the same time, the indirect spectrum extracted from the 2D ^27^Al­{^29^Si} D-HMQC data of Si10–Al_2_O_3–600_ evidences relatively high signal intensities around −85 ppm, corresponding to (AlO)_(4–*p*)_Si­(OSi)_*p*_ or (AlO)_(3–*p*)_SiOH­(OSi)_*p*_.^70^ The presence of the latter sites in Si10–Al_2_O_3–600_ is also evidenced by ^29^Si­{^27^Al} D-RINEPT, as the intensity around −85 ppm in the ^29^Si­{^27^Al} D-RINEPT spectrum is intermediate between the intensities at −85 ppm of the ^27^Al­{^29^Si} D-HMQC and ^29^Si­{^1^H} DNP SENS spectra, explained by the used CPMG acquisition scheme for signal-to-noise enhancement, which increases to a higher extent intensities of sites with long transverse relaxation times such as Si­(OSi)_4_ relative to silanol sites.^71^

Two-dimensional Al/Si correlations presented in Figure 6e,f reveal a more disordered nature of Si sites in Si10–Al_2_O_3–600_ than in Si1–Al_2_O_3–600_, as the former contains a broader range of silicon environments, spreading the 2D line shapes along the ^29^Si dimension. For Si1–Al_2_O_3–600_, there is no preference of Si to a specific Al coordination (i.e., ^[4]^Al, ^[5]^Al, or ^[6]^Al) in (AlO)_(4–*p*)_Si­(OSi)_*p*_ or (AlO)_(3–*p*)_SiOH­(OSi)_*p*_; in other words, all Si sites are close to Al sites in all three coordination geometries. This does not appear to be the case for Si10–Al_2_O_3–600_ since the maxima of the 2D lines are not at the same position on the ^29^Si dimension, yet the low S/N of those 2D lines prevents a more detailed analysis.

## Discussion

In a previous report, it was shown that the deposition of one ALD cycle of trimethylaluminum (3.4 wt % Al deposited) onto amorphous dehydroxylated silica generates a higher amount of strong BAS than after 5 or 10 ALD cycles, i.e., strong BAS reside primarily at the AlO_*x*_/SiO_2_ interface rather than on the subsequently grown AlO_*x*_ layers.^24^ In contrast, the present results reveal that the deposition of SiO_*x*_ by ALD onto dehydroxylated crystalline alumina requires 5–10 ALD cycles (5.9–9.9 wt % Si) for an appreciable amount of strong BAS to form. The relatively high amount of deposited Si required to yield strong BAS is likely related to the crystallinity of alumina, as similar findings have been reported when comparing materials (after calcination) obtained by grafting molecular Al precursors onto amorphous silica and Si precursors onto crystalline alumina.^25^ Specifically, it was found that grafting Al precursors onto silica, followed by calcination, preferentially formed ^[4]^Al–O–^[4]^Si linkages (a ^[4]^Al_(Si)_ site), which has been linked to strong BAS.^25^ It has been argued that the higher covalency of the Al–O–Si bonding relative to the more ionic Al–O–Al bonding causes an increase in the relative ratio of ^[4]^Al/^[6]^Al sites, as Al sites prefer a ^[4]^Al coordination geometry in more covalent environments.^25^ Our work estimates the relative ratio of ^[4]^Al:^[5]^:Al:^[6]^Al sites in the vicinity of Si atoms in Si1- and Si10–Al_2_O_3–600_ as 58:24:18 and 69:13:18, respectively, implying that a more significant amount of deposited Si increases the relative fraction of ^[4]^Al sites around Si, which is associated with the emergence of strong BAS and an increase in strength of LAS.^21^ Considering that TEM reveals a homogeneous, several nanometers thin overlayer of SiO_*x*_ on Si10–Al_2_O_3_, and at the same time, there is an appreciable amount of strong BAS together with strong and medium LAS in this material, and no weak LAS of alumina, the diffusion of Al^3+^ from the SiO_*x*_/Al_2_O_3_ interface into the surface (layer) of the deposited SiO_*x*_ is very likely.

Interestingly, in both Si1 and Si10–Al_2_O_3–600_, the intensity of ^[6]^Al sites around Si atoms in the ^27^Al­{^29^Si} SR4_1_^2^ D-HMQC spectrum is higher than that of the ^[4]^Al and ^[5]^Al sites when using a short recoupling time of 1.5 ms. This result indicates that also for Si10–Al_2_O_3–600_, the interface between SiO_*x*_ and Al_2_O_3_ contributes most to the Al signal intensity, and the amount of isolated ^[4]^Al_(Si)_ sites within the ALD-grown layer and on its surface is relatively low. This is indeed consistent with the largely decreased intensity of Py–LAS bands in the Py-FTIR experiment on Si10–Al_2_O_3–600_ compared to Si1–Al_2_O_3–600_. Noteworthy, the presence of a submonolayer SiO_*x*_ in Si1–Al_2_O_3–600_ resulted in the increase of the strength of all LAS (i.e., weak, medium, and strong LAS of alumina). Interestingly, ^15^N-Py DNP SENS distinguishes two strong BAS in Si5- and Si10–Al_2_O_3–600_ materials. These two Bro̷nsted sites may be due to pseudobridging silanols and zeolite-like BAS, as reported for ASA.^28,29^ The proposed processes prevailing in the ALD-prepared materials and key experimental observations are summarized in Scheme 1.

**Scheme 1:**
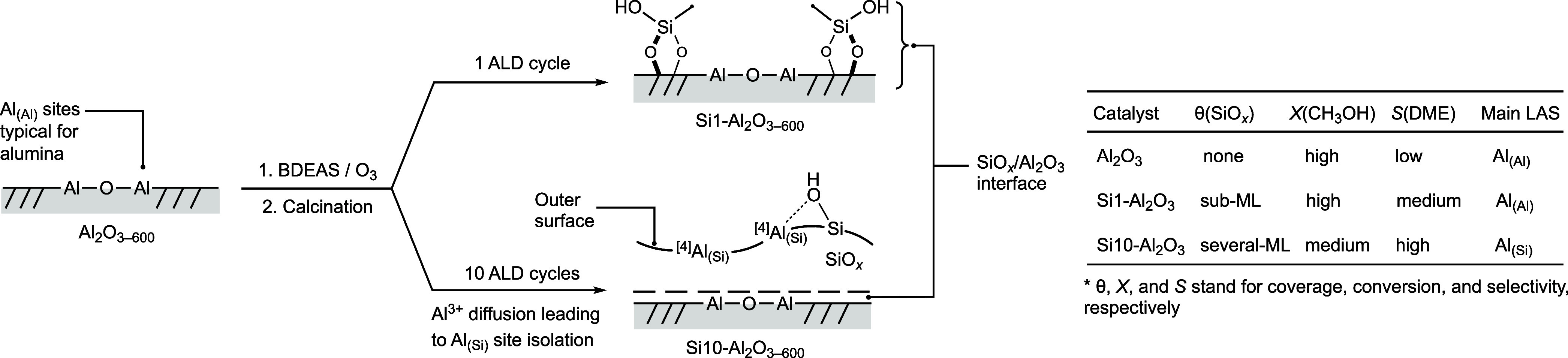
Summary of Key Structural and Surface Properties Distinguishing Al_2_O_3–600_ and Si1–Al_2_O_3–600_ from Si5- and Si10–Al_2_O_3–600_ in Methanol Dehydration^a^

a For Si1–Al_2_O_3–600_, the SiO_*x*_/Al_2_O_3_ interface is the particles’ outer surface.

Next, we aim to correlate insights into the local structure of Si and Al sites and surface acidity with methanol-to-DME conversion at 250 °C and DME selectivity at 450 °C. Alcohol dehydration on γ-Al_2_O_3_ has been reported to proceed on a Lewis acid – Lewis base pair.^72,73^ More specifically, an Al–O–Al­(OH) site has been proposed for the dehydration reaction, which transforms adsorbed methanol into an OCH_3_* species and, through interaction with a second methanol molecule, to DME.^74^ This mechanism is presented in Scheme 2a. Notably, the pathway requires an Al_(Al)_ site to activate the second methanol molecule on the neighboring (to the formed OCH_3_* species) LAS, facilitating the methoxy group attack and leading to the formation of DME. However, diverging results have been reported regarding the role of the strength of the specific Lewis acid sites. An early report on 1-butanol dehydration on Al_2_O_3_ suggested weak and mild LAS as the active dehydration sites,^75^ which was subsequently confirmed for the conversion of methanol-to-DME on γ-Al_2_O_3_.^76^ That being said, strong LAS on silica–alumina catalysts and strong BAS on zeolite catalysts have also been discussed as the active centers for ethanol and methanol dehydration, respectively.^74,77^

**Scheme 2:**
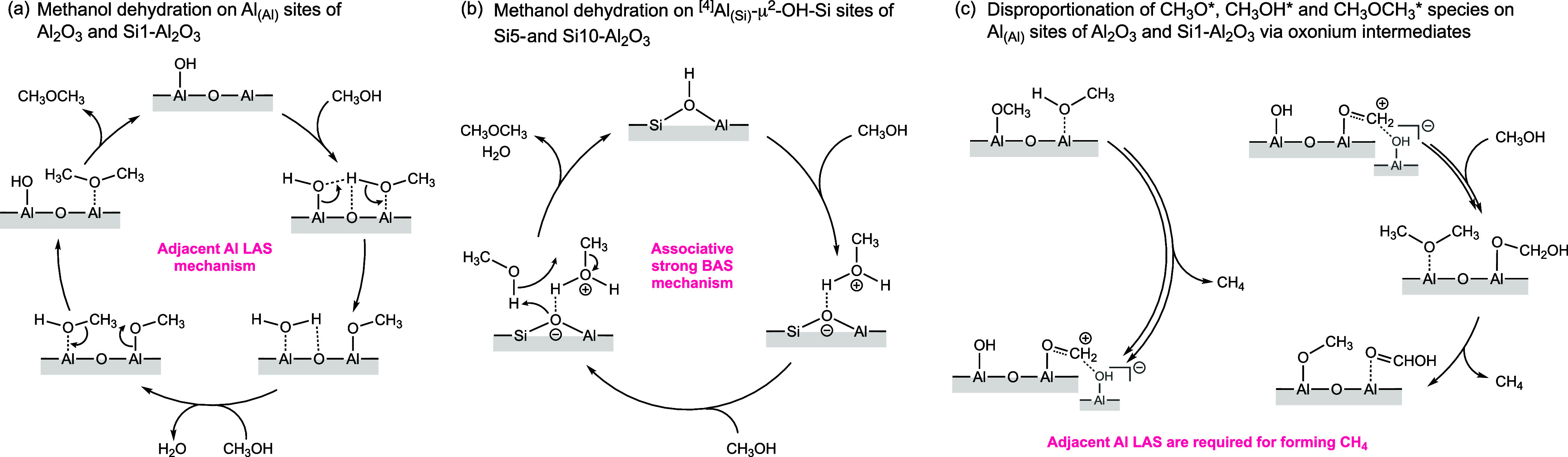
Proposed Mechanisms for the Methanol-to-DME Dehydration on (a) Al_(Al)_ Weak LAS of Al_2_O_3–600_ and Si1–Al_2_O_3–600_, (b) Strong BAS of Si5- and Si10–Al_2_O_3–600_, and (c) Key Steps for the DME Decomposition to Methane on the Al_(Al)_ Site of Al_2_O_3_

Considering these previous reports, the higher methanol conversion at 250 °C on Al_2_O_3–600_ and Si1–Al_2_O_3–600_ and the lower conversion on Si5 and Si10–Al_2_O_3_ catalysts correlates with the amount of Al_(Al)_ LAS, i.e., the proposed active sites for the mechanism presented in Scheme 2a. Both the total amount of LAS and the amount of Al_(Al)_ LAS decrease with increasing Si loading, leading to a simultaneous increase in strong BAS. While an increasing quantity of Si deposited leads to a higher relative fraction of ^[4]^Al_(Si)_ sites, which are stronger LAS than those in Al_2_O_3–600_, there is a concurrent decrease in the total amount of LAS due to the coverage of the Al^3+^ sites of alumina with a SiO_*x*_ layer that is offset only partially by the diffusion of Al^3+^ into the SiO_*x*_ overlayer in Si5- and Si10–Al_2_O_3–600_. The presence of weak LAS in Al_2_O_3–600_ and Si1–Al_2_O_3–600_, ascribed to ^[5]^Al_(Al)_ sites of alumina,^25,54^ indeed correlates with the higher methanol conversion of Al_2_O_3–600_ and Si1–Al_2_O_3–600_ at 250 °C, while the lack of such weak LAS and isolation of Al^3+^ sites in Si5- and Si10–Al_2_O_3–600_ correlates with their lower methanol conversion at 250 °C. As weak Al_(Al)_ LAS are not found in Si5- and Si10–Al_2_O_3–600_, it is conceivable that the latter two catalysts convert methanol to DME via a strong BAS mechanism presented in Scheme 2b. Furthermore, the structural differences as a function of Si deposition as discussed above, also explain the trend in DME decomposition at 450 °C. As the formation of DME from methanol, also the decomposition of DME has been suggested to involve two adjacent Al atoms, i.e., Al_(Al)_ sites.^45^ Consistent with this hypothesis, in our work, the weak or medium strength Al_(Al)_ LAS of Al_2_O_3–600_ and Si1–Al_2_O_3–600_ were found to decompose DME to CH_4_ through the steps depicted in Scheme 2c. At the same time, strong LAS such as ^[4]^Al_(Si)_ in Si5- and Si10–Al_2_O_3–600_ or BAS do not drive this reaction due to the lack of two adjacent Al atoms, confirming site isolation of Al^3+^ sites in these materials.

## Conclusion

This study provides an atomic-scale characterization of ALD-derived silica layers on partially dehydroxylated Al_2_O_3_, offering insight into the local structures at the interfacial sites that control surface acidity and the catalytic activity of the materials in methanol dehydration. Specifically, it was found that Al_(Al)_ Lewis sites drive methanol dehydration to DME at 250 °C in Al_2_O_3–600_ and Si1–Al_2_O_3–600_. Covering such Al_(Al)_ sites with silica overlayers and the diffusing of Al^3+^ within the grown layer creates strong LAS and BAS containing ^[4]^Al_(Si)_ sites. Such isolated ^[4]^Al_(Si)_ surface sites are observed in Si5- and Si10–Al_2_O_3–600_ but are largely absent in Si1–Al_2_O_3–600_, i.e., at the SiO_*x*_/Al_2_O_3_ interface. The isolated ^[4]^Al_(Si)_ LAS are less active in methanol dehydration to DME at 250 °C relative to the Al_(Al)_ sites of alumina. However, ^[4]^Al_(Si)_ sites, unlike Al_(Al)_ sites, do not readily decompose DME to CH_4_ at 450 °C. Our structural analysis reveals the evolution of surface acidity with increasing quantities of SiO_*x*_ being deposited by ALD, underscoring the critical role of such silica–alumina overlayers in determining the catalytic properties in methanol dehydration to DME and the decomposition of DME to methane. This insight enhances our atomic-level understanding of surface and interfacial properties, leading ultimately to the design of more efficient heterogeneous catalysts.

## Supplementary Material



## Notes

Experimental procedures; additional data analyses; N_2_ adsorption and desorption isotherms; IR spectra; XRD patterns; TEM and HAADF-STEM images; EDX maps; product selectivity plot; NH_3_ TPD deconvolution plots; CO–DRIFTS spectra; simulations of ^27^Al­{^29^Si} SR4_1_^2^ D-HMQC spectra; Raman spectra; tabulated results of Si loading, BET surface area, pore volume, and pore diameter; fitting parameters of the ^29^Si and ^15^N DNP SENS data; results of the catalytic tests; and quantification of Py-FTIR spectra and NH_3_ TPD (PDF)
