# Supplementary material for: Local Structure and Surface Acidity of Overlayers Prepared by Atomic Layer Deposition of Silica on Alumina
Source: Chem Mater. 2025 Sep 30;37(19):7974–86. doi: 10.1021/acs.chemmater.5c01832 (PMC12529904; doi:10.1021/acs.chemmater.5c01832)
Supplement: Supplementary file 1 [file cm5c01832_si_001.pdf]

Supporting information

Local Structure and Surface Acidity of Overlayers Prepared by Atomic Layer Deposition of Silica on Alumina

Melis Yarar,[1] Zixuan Chen,[1] Diana Piankova,[1] Matthias Becker,[1] Alexander V. Yakimov,[2] Christophe Copéret,[2] Pierre Florian,[3*] Christoph R. Müller,[1*] Alexey Fedorov[1*]

[1] Department of Mechanical and Process Engineering, ETH Zürich, CH-8092 Zürich, Switzerland
[2] Department of Chemistry and Applied Biosciences, ETH Zürich, CH-8093 Zürich, Switzerland
[3] CNRS, CEMHTI UPR3079, Université d'Orléans, Orléans 45071, France

Emails:
pierre.florian@cnrs-orleans.fr
muelchri@ethz.ch
fedoroal@ethz.ch

Table of Contents

Experimental Details **4**

Synthesis of materials *4*

Characterization *4*

Catalytic testing *7*

Supporting Figures **8**

Supporting Tables **18**

References **21**

FIGURES

Figure S1. FTIR spectra of air-exposed Al_2O_3 and dehydroxylated Al_2O_{3-600} in black and blue, respectively........ 8
Figure S2. FTIR spectra of Al_2O_{3-600} and the materials obtained after 5 and 20 pulses of BDEAS on Al_2O_{3-600} without ozone pulses compared to BDEAS1-Al_2O_{3-600} and Si1-Al_2O_{3-600} (black, purple, blue, red, and green traces, respectively). 8
Figure S3. N_2 adsorption and desorption isotherms of Al_2O_{3-600}, Si1,5,10-Al_2O_{3-600}, and SiO_{2-600} air-exposed materials. 9
Figure S4. BJH pore size distribution obtained from the N_2 isotherms of Al_2O_{3-600}, Si1,5,10-Al_2O_{3-600}, and SiO_{2-600} air-exposed materials........ 10
Figure S5. XRD patterns of Al_2O_{3-600}, Si1,5,10-Al_2O_{3-600}, and SiO_{2-600} recorded in air. 11
Figure S6. Overlap of the elemental maps of Al K (in green) and Si K (in red) of air-exposed (a) Si1-Al_2O_{3-600}, (b) Si5-Al_2O_{3-600}, and (c) Si10-Al_2O_{3-600}........ 11
Figure S7. Overview TEM images of (a) Al_2O_{3-600} and (b) Si10-Al_2O_{3-600}. 11
Figure S8. (a) HAADF-STEM image of the area of Si10-Al_2O_{3-600} studied by EDX, (b) EDX spectrum, (c, d, e) elemental maps of Al K, Si K and their overlap, respectively. (f) EDX profile analysis performed along the line drawn in (a), (g) HAADF-STEM image of the area of Si10-Al_2O_{3-600} studied by EDX, (h) overlap of the EDX Al K and Si K elemental maps, (i) EDX profile analysis performed along the line drawn in (g)........ 12
Figure S9. Selectivity to DME, and methane and carbon balance as a function of methanol conversion for (a) Al_2O_{3-600}, (b) Si1-Al_2O_{3-600}, (c) Si5-Al_2O_{3-600}, and (d) Si10-Al_2O_{3-600}. 13
Figure S10. Average of 3 independent NH_3-TPD runs of Al_2O_{3-600} showing experimental reproducibility. 13
Figure S11. The deconvolution of the NH_3-TPD peaks of (a) Al_2O_{3-600}, (b) Si1-Al_2O_{3-600}, (c) Si5-Al_2O_{3-600}, (d) Si10-Al_2O_{3-600}. 14
Figure S12. Py-FTIR spectra of (a) SiO_{2-600} in the 1710-1390 cm^{-1} range, and (b) ^{15}N-Py and Py-FTIR spectra of Al_2O_{3-600} and Si1-, Si5, and Si10-Al_2O_{3-600} in the 1470-1430 cm^{-1} range. 14
Figure S13. CO-DRIFTS profiles of Al_2O_{3-600}, Si1-, Si5, Si10-Al_2O_{3-600}, and SiO_{2-600} with increasing CO pressure........ 15

Figure S14. CO-DRIFTS profiles of Al_2O_{3-600}, Si1, Si5, Si10-Al_2O_{3-600}, and SiO_{2-600} for decreasing CO pressure........16
Figure S15. Simulations of $^{27}Al\{^{29}Si\}$ SR4_1^2 D-HMQC spectra of (a) Si1-Al_2O_{3-600} and (b) Si10-Al_2O_{3-600} using 6.0 ms for recoupling time........17
Figure S16. (a) Raman spectrum of post-reaction Al_2O_{3-600} exposed to air and (b) attenuated total reflectance IR spectra of the fresh and post-reaction Al_2O_{3-600}, exposed to air. 17

TABLES

Table S1. BET surface area, pore volume, and pore diameter of Al_2O_{3-600}, Si1,5,10-Al_2O_{3-600}, and SiO_{2-600} ambient air-exposed materials........18
Table S2. Fitting parameters of the ^{29}Si DNP SENS data........18
Table S3. Results of the catalytic tests. 19
Table S4. The amount of LAS calculated from the Py-FTIR spectra and the total number of acidic sites calculated from ammonia TPD profiles........20
Table S5. Parameters obtained by fitting the ^{15}N DNP SENS data. 20

Experimental Details

Synthesis of materials

The alumina powder (Puralox, SBa 200, Sasol) was used as a substrate for the atomic layer deposition (ALD) of silica. Before deposition, alumina was agglomerated by wetting with distilled water and kept at 120 °C for 2 days. The resulting agglomerates were sieved, and a 200-450 µm size fraction was collected. The sieved alumina was calcined overnight at 500 °C under static air and then dehydroxylated overnight at 600 °C at ca. 10^{-5} mbar. The resulting material is denoted as Al_2O_{3-600} and is expected to have an OH density of approximately 1 OH nm^{-2}.[1] The dehydroxylated Aerosil silica, SiO_{2-600}, was prepared following the above procedure and used as a reference material.

The ALD of silica was performed using a Picosun R-200 system enclosed inside an MBraun glovebox (O_2, H_2O < 1-2 ppm). N_2 (99.999 %) was used as the carrier and purge gas. A typical experiment involved the ALD of bis(diethylamino)silane (BDEAS, Pegasus Chemicals) on 300 mg of Al_2O_{3-600}, heated to 300 °C. The vessel containing the BDEAS precursor was heated to 60 °C. Ozone was used as the oxidant (estimated concentration of 10 % w/w, 140 g Nm^{-3}). The optimization of the ALD recipe included pulsing BDEAS on Al_2O_{3-600} until the subsequent pulse resulted in no significant change in the hydroxyl and C–H regions of the resulting BDEAS-Al_2O_{3-600} as probed by Fourier-transform infrared spectroscopy (FTIR). Under the optimized conditions one ALD cycle consisted of 20 pulses of BDEAS (0.4 s pulse time) followed by 10 ozone pulses (5 s pulse time), separated by a 15-second N_2 purge. Either 1, 5, or 10 deposition cycles were performed. The resulting materials are denoted BDEAS1-Al_2O_{3-600}, BDEAS5-Al_2O_{3-600}, or BDEAS10-Al_2O_{3-600}, respectively. The as-deposited materials were calcined under a flow of synthetic air (50 mL min^{-1}) at 600 °C for 3 h, resulting in materials denoted as Si1-Al_2O_{3-600}, Si5-Al_2O_{3-600}, and Si10-Al_2O_{3-600}.

Characterization

Elemental analysis. The Si weight loading of each prepared material was obtained using inductively coupled plasma optical emission spectroscopy (ICP-OES) performed at Mikroanalytisches Labor Pascher, Remagen.

Surface area and pore volume characterization with N_2 physisorption. N_2 adsorption/desorption (Quantachrome NOVA 4000e) applying the Brunauer-Emmet-Teller (BET) and Barrett-Joyner-Halenda (BJH) models was used to determine the surface area and pore volume of the materials.

Powder X-ray diffraction (XRD). A PANalytical Empyrean X-ray diffractometer equipped with a Bragg-Brentano HD mirror, Cu Kα radiation (λ = 1.5418 Å) was used to collect XRD data of the studied materials. The following experimental parameters were used: 2Θ° = 5-100°; step size = 0.0167; scan step time = 70.485 s.

Scanning/Transmission electron microscopy (TEM and STEM). Scanning/Transmission electron microscopy was performed on a double Cs-corrected transmission electron microscope, JEM-ARM300F Grand ARM "Vortex," operated at 300 kV. This microscope has two large-area SDD EDX detectors with a 100 mm^2 active area. The specimens were prepared in the air by directly dipping the Au grids coated with lacey carbon into the powder of the ALD-made materials.

Raman spectroscopy. Raman spectrum of the post-reaction alumina material was collected on a Thermo Scientific DXR Raman spectrometer equipped with a 532 nm laser in the range of 3720–100 cm^{-1} with a resolution of 6 cm^{-1}.

Attenuated total reflectance infra-red spectroscopy (ATR IR). The ATR IR spectrum of the post-reaction alumina material was collected on a Nicolet iS50 spectrometer equipped with a Smart iTR™ ATR sampling accessory, in the range of 4000–650 cm^{-1} with a resolution of 4 cm^{-1}. The number of scans collected was 32.

Transmittance Fourier transform infrared spectroscopy (FTIR). The collection of the FTIR spectra of the dehydroxylated materials was performed on an Alpha II spectrometer (Bruker) inside an MBraun glovebox (O_2, H_2O < 1-2 ppm). Material transfers were performed without exposure to ambient air. All absorbance FTIR spectra presented in this work are normalized to the pellet mass.

Surface acidity study with pyridine. Pyridine (Py) and ^{15}N-Pyridine (99% isotopic enrichment, ^{15}N-Py, CortecNet Corp.) were dried over CaH_2 at 60 °C for two days and degassed by three freeze-pump-thaw cycles before use. The materials were evacuated (ca. 10^{-5} mbar) and then exposed to either Py or ^{15}N-Py vapor at ambient temperature for one minute, followed by outgassing at 150 °C for 1 hour with a ramping rate of 5 °C min^{-1} and a pressure of ca. 10^{-5} mbar. The FTIR spectra of the outgassed specimen were collected using an Alpha II spectrometer (Bruker) inside an N_2-filled glovebox at room temperature, using self-supporting pellets of 7 mm in diameter. The amount of the sample per unit area of the pellets was ca. 20-40 mg cm^{-2}. The spectra were collected with a resolution of 4 cm^{-1}, and 16 scans were averaged. The amount of Lewis acidic sites was calculated by using a modified Beer-Lambert-Bouguer law and the FTIR spectrum of the nonlabelled pyridine adsorbed materials:

$$n_{py} = \frac{A \times S}{\varepsilon_{\mathrm{Py-L}}},$$

where A is an area of the FTIR peak of the nonlabelled pyridine at 1443 cm^{-1} (the integration range for the calculation of the peak area was 1465-1435 ± 8 cm^{-1}), S is the cross-sectional area of the pellet, and $\varepsilon_{\mathrm{Py-L}}$ is the molar absorption (extinction) coefficient of pyridine adsorbed on Lewis acidic sites, taken as 1.87 cm μmol^{-1} according to the literature.[2]

Diffuse reflectance infrared Fourier transform spectroscopy (DRIFTS). Experiments using carbon monoxide as a probe molecule (CO-DRIFTS) were conducted on a Nicolet 6700 FTIR spectrometer equipped with a Praying Mantis™ low-temperature reaction cell. The specimens were pressed into ca. 10 mg pellets in ambient air. The pellets were placed in the DRIFTS cell and dehydroxylated overnight under ca. 10^{-3}–10^{-4} mbar at a temperature of 600 °C. Dehydroxylated pellets were cooled down to ca. –120 °C, and background spectra were collected under a vacuum. CO gas was pulsed inside the cell at increments of ca. 7 mbar until ca. 100 mbar was reached. After 100 mbar of CO pressure was reached, the cell was outgassed, and DRIFTS spectra were recorded at approximately 80, 60, 40, 20, and 5 mbar during the outgassing.

Ammonia temperature programmed desorption (NH_3-TPD). A quantification of surface acid sites was performed using NH_3-TPD experiments conducted on a Micromeritics AutoChem 2920 instrument. Ca. 50 mg of a specimen was placed in a quartz reactor between two layers of quartz wool. The materials were pretreated by heating to 600 °C under Ar flow (20 ml min^{-1}) and holding at this temperature for 60 minutes. Subsequently, it was cooled to 120 °C under Ar flow. Ammonia adsorption was performed at 120 °C with a 5% NH_3/He gas mixture (50 ml min^{-1}, 30 min). Physiosorbed NH_3 was removed under a He flow (20 ml min^{-1}) at 120 °C for 2 h. Subsequently, NH_3-TPD was performed under a He flow (20 ml min^{-1}) with a temperature ramp of 10 °C min^{-1} while heating to 600 °C. The off-gas was analyzed with an online mass spectrometer (MKS Cirrus2). The calibration of the NH_3 MS signal was done by pulsing 5% NH_3/He through a loop of a known volume (0.4943 cm^{-3}) at a constant

temperature (110 °C) and atmospheric pressure. The pulses were repeated 48 times and averaged. The integrated mass spectrum area at m/z = 16 was used for quantification (to avoid errors associated with the signal at m/z = 17, which contains a contribution from water fragmentation). A calibration factor was calculated as moles of NH_3 per unit area of the m/z = 16 MS signal. The quantification of desorbed NH_3 during NH_3-TPD was performed by multiplying the integrated area of the m/z = 16 signal by the calibration factor. One molecule of NH_3 was assumed to interact with one acid site. To assess the experimental errors, three independent NH_3-TPD experiments were conducted with Al_2O_{3-600}, and the averaged results and error bars are given in Figure S10.

^{27}Al{^{29}Si} dipolar heteronuclear multiple-quantum coherence (D-HMQC). The SR4_1^2 D-HMQC experiments,[3] obtained on a Bruker Neo 17.6 T spectrometer operating at ^{27}Al and ^{29}Si frequencies of 195.4 MHz and 149.0 MHz, respectively, used 3.2 mm rotors spinning at 20 kHz in a triple resonance probe. ^{27}Al and ^{29}Si radio-frequency fields were set to 10 kHz (corresponding to a T90 selective pulse of 7 μs) and 30 kHz, respectively, with a double frequency sweep (DFS) applied to ^{27}Al prior to detection (i.e., a 1.2 ms pulse sweeping from +/– 1400 kHz to +/– 150 kHz at a radio-frequency field of 30 kHz). A recycle delay of 2 s (Si1-Al_2O_{3-600}) and 10 s (Si10-Al_2O_{3-600}) was used to avoid signal saturation with, for the 2D correlations, 9 (Si1-Al_2O_{3-600}) and 5 (Si10-Al_2O_{3-600}) indirect increments of 100 μs acquired using a States-TPPI procedure along with 6656 (Si1-Al_2O_{3-600}) and 1680 (Si10-Al_2O_{3-600}) transients accumulated for each slice (for a total duration of approximately 3 days for Si10-Al_2O_{3-600} and 2 days for Si10-Al_2O_{3-600}). The 2D correlations were performed with a recoupling time of 6 ms. Variable recoupling time 1D experiments were obtained by accumulating 16384 scans with a 2 s recycled delay and simulated using the DMFit software,[4] with a so-called "Czjzek" or "Gaussian Isotropic Model" lineshape.[5]

^{29}Si{^{27}Al} dipolar-mediated refocused insensitive nuclei enhanced by polarization transfer (D-RINEPT). The SR4_1^2 D-RINEPT experiments have been obtained on a Bruker Neo 17.6 T spectrometer operating at ^{27}Al and ^{29}Si frequencies of 195.4 MHz and 149.0 MHz, respectively, and using 3.2 mm rotors spinning at 15 kHz in a triple resonance probe. ^{27}Al and ^{29}Si radio-frequency fields were set to 10 kHz (corresponding to a T90 selective pulse of 7 μs) and 40 kHz, respectively, with DFS applied to ^{27}Al before transfer to ^{29}Si, which is detected using a CPMG train of 200 echoes separated by 4 ms. The number of accumulated transients using a recoupling time of 2 ms and a recycle delay of 2 s was 32768 for Si1-Al_2O_{3-600} and 2048 for Si10-Al_2O_{3-600}.

^{29}Si dynamic nuclear polarization surface enhanced NMR spectroscopy (^{29}Si DNP SENS). The DNP SENS measurements were performed for the materials Si1-, Si5-, ans Si10-Al_2O_{3-600} using a 16 mM TEKPol in the 1,1,2,2-tetrachloroethane (TEKPol TCE) solution. The materials (ca. 20 mg) were impregnated with 1 µL of TEKPol TCE. The impregnated materials were loaded into a 3.2 mm sapphire rotor inside an argon-filled glovebox (O_2, H_2O < 0.5 ppm) and sealed with a zirconia cap to minimize air exposure during transfer from the glovebox to the spectrometer. The spectra were collected on a Bruker 600 MHz spectrometer equipped with a gyrotron microwave source (395 GHz, output power of 6 – 10 W). A magic angle spinning (MAS) rate of 8 kHz was employed. DNP-enhanced ^{1}H-^{29}Si NMR spectra were recorded using a CP-MAS pulse sequence with ^{1}H excitation (96 kHz), contact time of 2 ms (48 kHz), followed by ^{1}H decoupling (62 kHz). Recovery delays were set to 1.3 times T_1, where T_1 was measured in saturation-recovery experiments for each sample. The number of scans in the DNP-enhanced ^{1}H-^{29}Si NMR spectra of Si1-Al_2O_{3-600}, Si5-Al_2O_{3-600}, and Si10-Al_2O_{3-600} were 11184, 15384, and 7312, respectively. The enhancement factors, obtained as the measured ^{1}H enhancement of the solvent, in DNP-enhanced ^{1}H-^{29}Si NMR spectra of Si1-Al_2O_{3-600}, Si5-Al_2O_{3-600}, and Si10-Al_2O_{3-600} were 27, 19, and 30, respectively. The spectra were fitted using the DMFit software.[4]

^{15}N DNP SENS. The DNP SENS measurements were performed using materials containing pre-adsorbed Py impregnated with TEKPol TCE, and handled as described above for the ^{29}Si DNP SENS measurements. DNP-enhanced ^{15}N NMR spectra were measured using a CP-MAS pulse sequence with ^{1}H excitation (83 kHz), contact time of 2 ms (23.5 kHz), followed by ^{1}H decoupling (62 kHz). The number of scans in the DNP-enhanced ^{1}H-^{15}N NMR spectra of Si1-Al_2O_{3-600}, Si5-Al_2O_{3-600}, Si10-Al_2O_{3-600}, and Al_2O_{3-600} were 1696, 448, 2040, and 824, respectively. The higher-frequency ^{13}C peak of adamantane at 38.4 ppm was used as a reference for the static magnetic field. The DNP buildup time (τDNP) was measured using a ^{1}H saturation-recovery experiment with microwaves turned on. The enhancement factors, obtained as the measured ^{1}H enhancement of the solvent, in DNP-enhanced ^{1}H-^{15}N NMR spectra of Si1-Al_2O_{3-600}, Si5-Al_2O_{3-600}, Si10-Al_2O_{3-600}, and Al_2O_{3-600} were 39, 37, 13, and 22, respectively.

Catalytic testing

The conversion of methanol to dimethyl ether (DME) was assessed on a Microactivity EFFI reactor (PID Eng&Tech). The catalysts were loaded into a quartz reactor (13 mm ID) under atmospheric conditions. The reactor was purged with N_2 for 30 min (45.5 ml min^{-1}) and heated to the starting reaction temperature of 150 °C (10 °C min^{-1}). A CH_3OH/N_2 reaction mixture was supplied to the reactor by passing an N_2 flow (10.0 ml min^{-1}, 1 bar) through a bubbler filled with liquid CH_3OH (Sigma-Aldrich, purity ≥ 99.9%). For the reaction tests, the flow was switched to 0.75% CH_3OH/N_2 (45.5 ml min^{-1}, 1 bar), and after one hour of time on stream (TOS), the reactor temperature was increased to 450 °C with increments of 100 °C, each held for 70 min. The products were analyzed with a gas chromatograph (CompactGC 4.0, Gas Analyser Solutions). The methanol conversion (X_{CH_3OH}), selectivity to products (S_i), carbon balance, and space-time yield of DME (STY_{DME}) were calculated as:

$$X_{\mathrm{CH_3OH}} = \left(1 - \frac{F_{\mathrm{CH_3OH,out}} \times N_{\mathrm{CH_3OH}}}{F_{\mathrm{CH_3OH,out}} \times N_{\mathrm{CH_3OH}} + \sum F_{\mathrm{i,out}} \times N_{i,out}}\right) \times 100\ \%$$

$$S_i = \frac{F_{\mathrm{i,out}} \times N_{i,out}}{\sum F_{\mathrm{i,out}} \times N_{i,out}} \times 100\ \%$$

$$Carbon\ balance = \frac{F_{\mathrm{CH_3OH,out}} \times N_{\mathrm{CH_3OH}} + \sum F_{\mathrm{i,out}} \times N_{i,out}}{F_{\mathrm{CH_3OH,in}} \times N_{\mathrm{CH_3OH}}} \times 100\ \%$$

$$STY_{DME}\left[\frac{g_{DME}}{gcat \times h}\right] = \frac{F_{DME,\mathrm{out}} \times MW_{DME}}{m_{cat}/1000} \times 60$$

Where $F_{\mathrm{CH_3OH,out}}$, $F_{\mathrm{i},out}$, $N_{i,out}$, MW_{DME}, m_{cat} represent the flow rate of methanol and that of carbon-containing products at the outlet (mol min^{-1}), the carbon number of the respective species, the molar mass of DME (46.07 g mol^{-1}), and the mass of the catalyst (mg), respectively. The factor 60 is used to convert minute-based flow rates into hourly-based flow rates.

Detailed results of the catalytic tests are given in Table S3. A control catalytic experiment with Al_2O_{3-600} was conducted, where the material was loaded in a glovebox and handled without exposure to ambient air. No major differences were observed between experiments with or without exposure to ambient air.

Supporting Figures

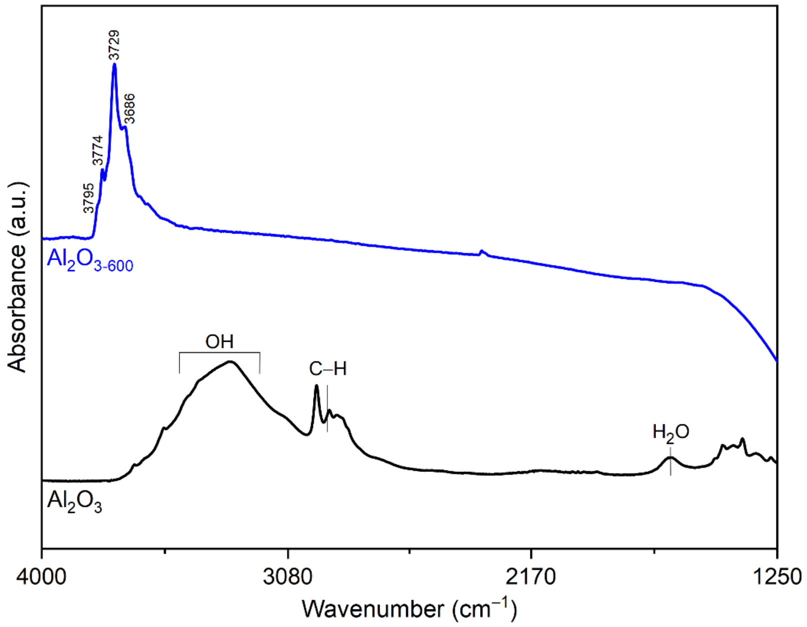

Figure S1. FTIR spectra of air-exposed Al_2O_3 and dehydroxylated Al_2O_{3-600} in black and blue, respectively.

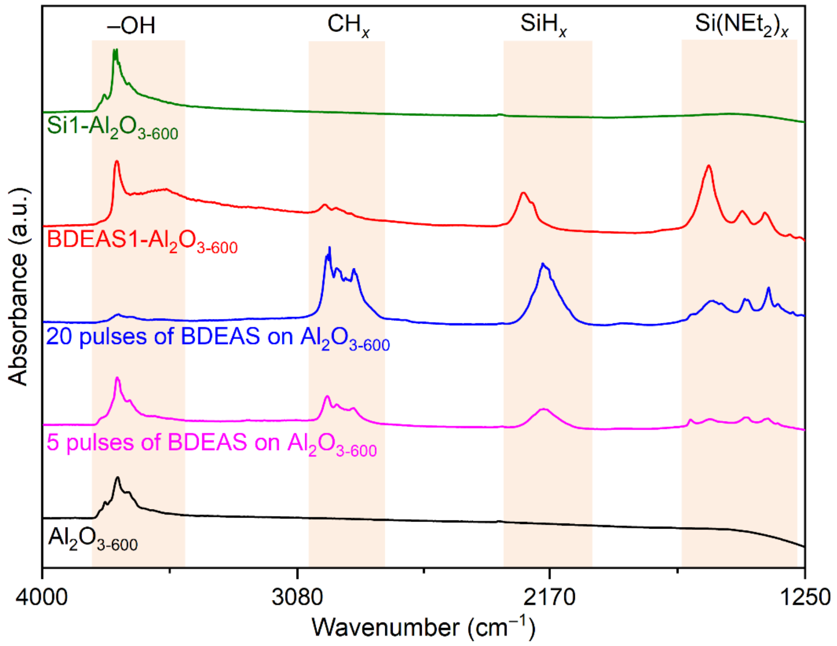

Figure S2. FTIR spectra of Al_2O_{3-600} and the materials obtained after 5 and 20 pulses of BDEAS on Al_2O_{3-600} without ozone pulses compared to BDEAS1-Al_2O_{3-600} and Si1-Al_2O_{3-600} (black, purple, blue, red, and green traces, respectively).

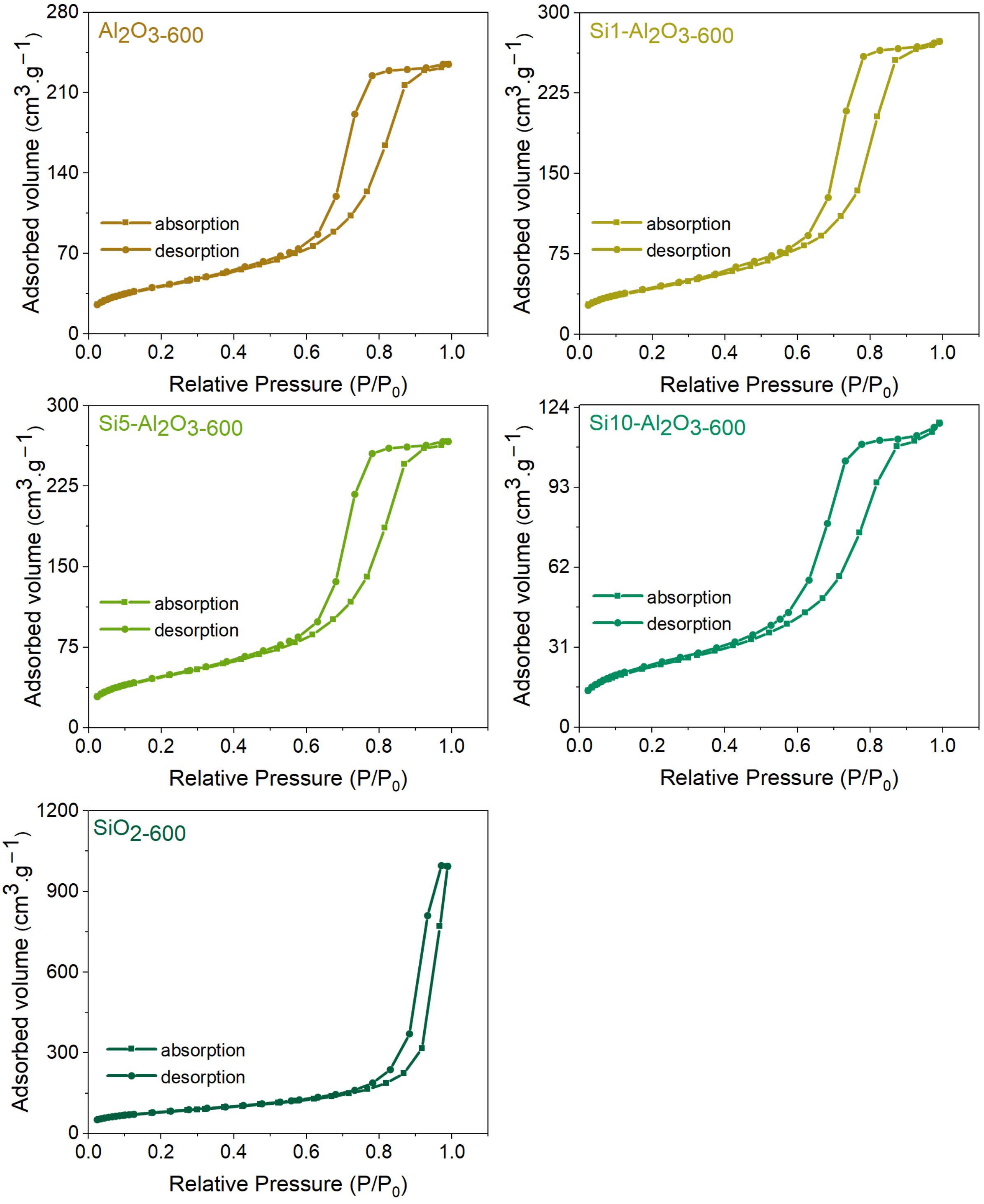

Figure S3. N_2 adsorption and desorption isotherms of Al_2O_{3-600}, Si1,5,10-Al_2O_{3-600}, and SiO_{2-600} air-exposed materials.

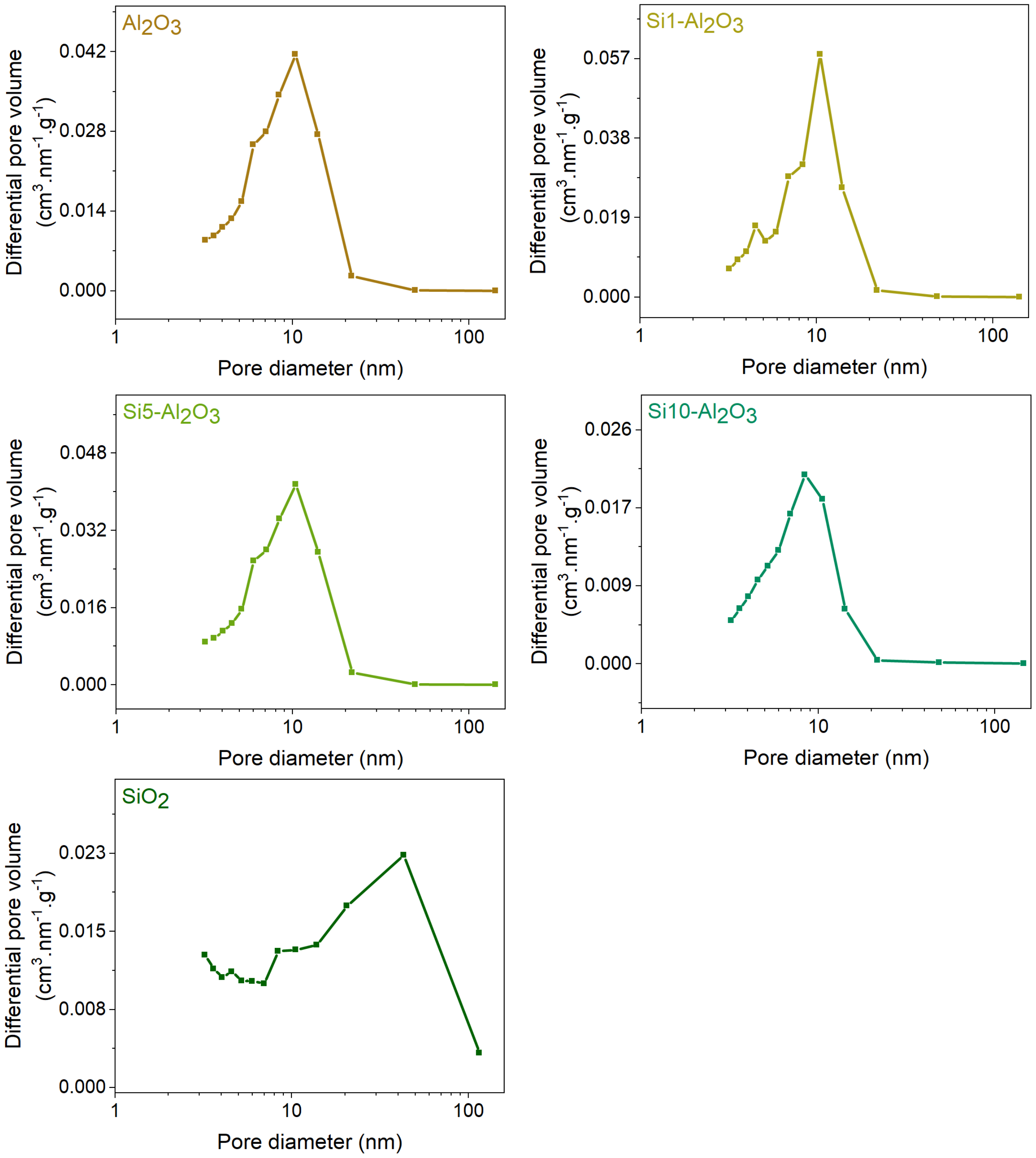

Figure S4. BJH pore size distribution obtained from the N_2 isotherms of Al_2O_{3-600}, Si1,5,10-Al_2O_{3-600}, and SiO_{2-600} air-exposed materials.

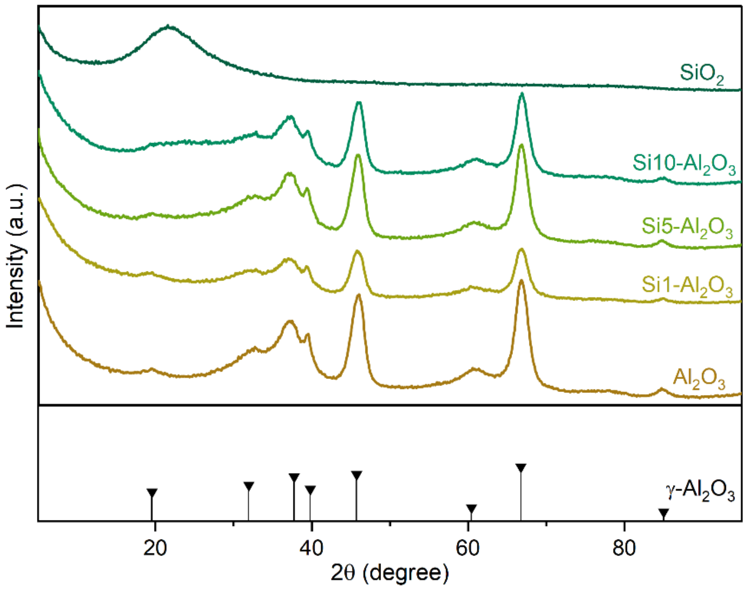

Figure S5. XRD patterns of Al_2O_{3-600}, Si1,5,10-Al_2O_{3-600}, and SiO_{2-600} recorded in air.

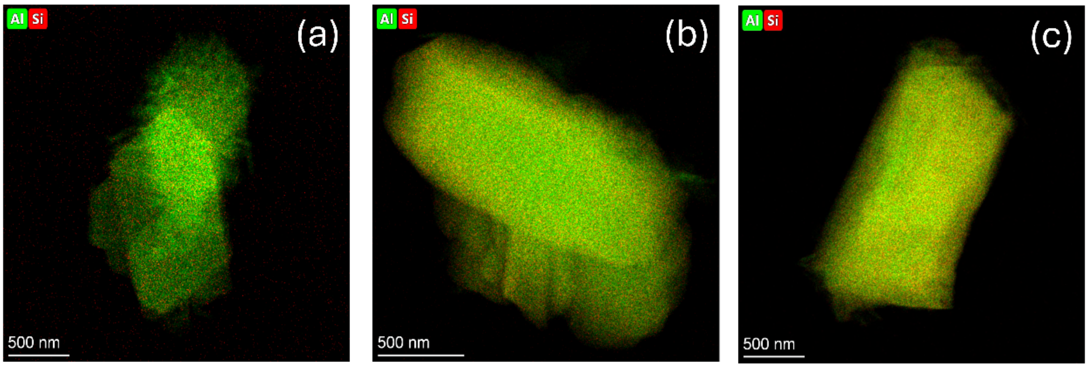

Figure S6. Overlap of the elemental maps of Al K (in green) and Si K (in red) of air-exposed (a) Si1-Al_2O_{3-600}, (b) Si5-Al_2O_{3-600}, and (c) Si10-Al_2O_{3-600}.

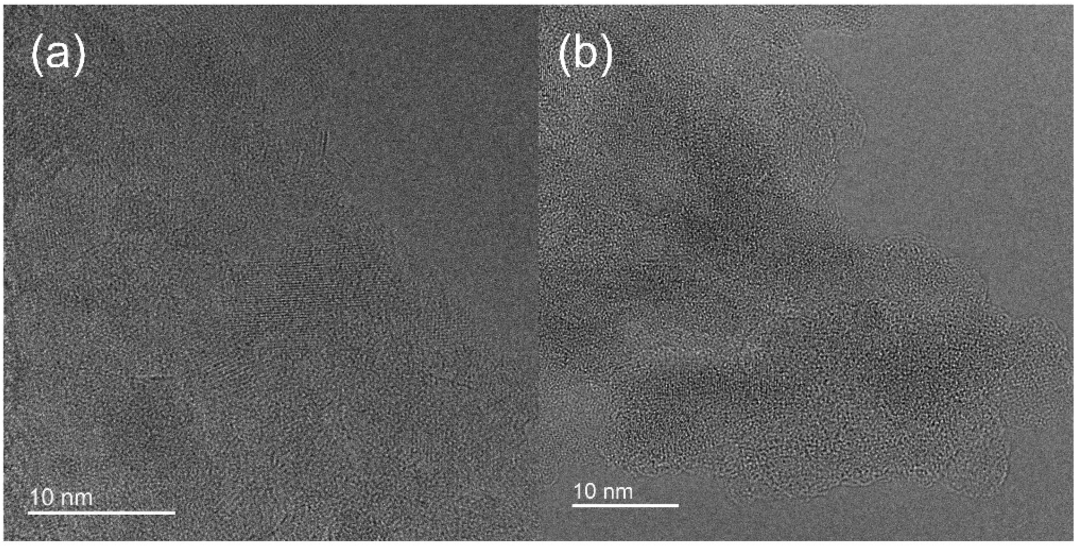

Figure S7. Overview TEM images of (a) Al_2O_{3-600} and (b) Si10-Al_2O_{3-600}.

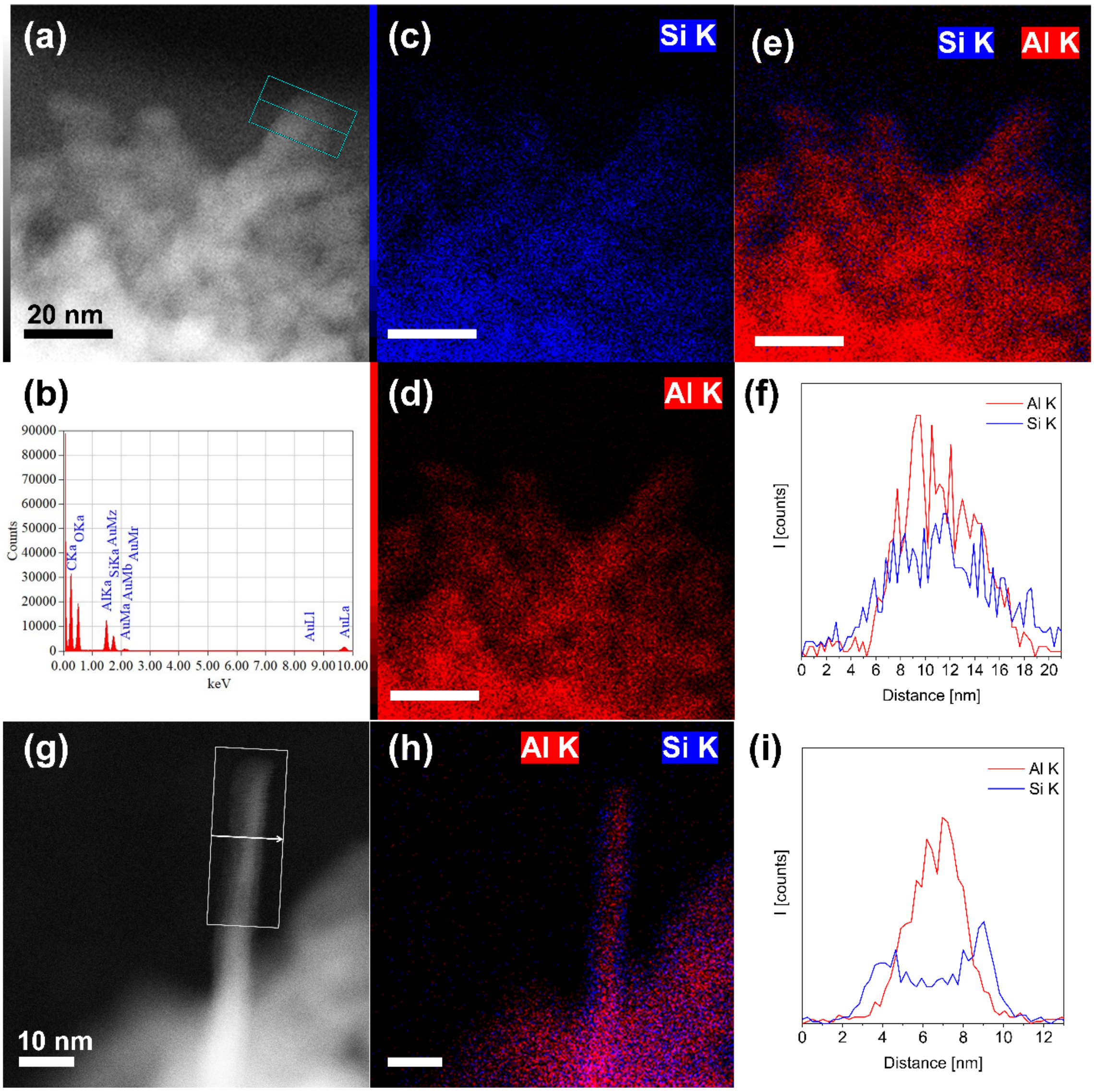

Figure S8. (a) HAADF-STEM image of the area of Si10-Al_2O_{3-600} studied by EDX, (b) EDX spectrum, (c, d, e) elemental maps of Al K, Si K and their overlap, respectively. (f) EDX profile analysis performed along the line drawn in (a), (g) HAADF-STEM image of the area of Si10-Al_2O_{3-600} studied by EDX, (h) overlap of the EDX Al K and Si K elemental maps, (i) EDX profile analysis performed along the line drawn in (g).

The EDX profile (line scan) analysis confirms more Si at the edges of alumina crystallites than in the bulk.

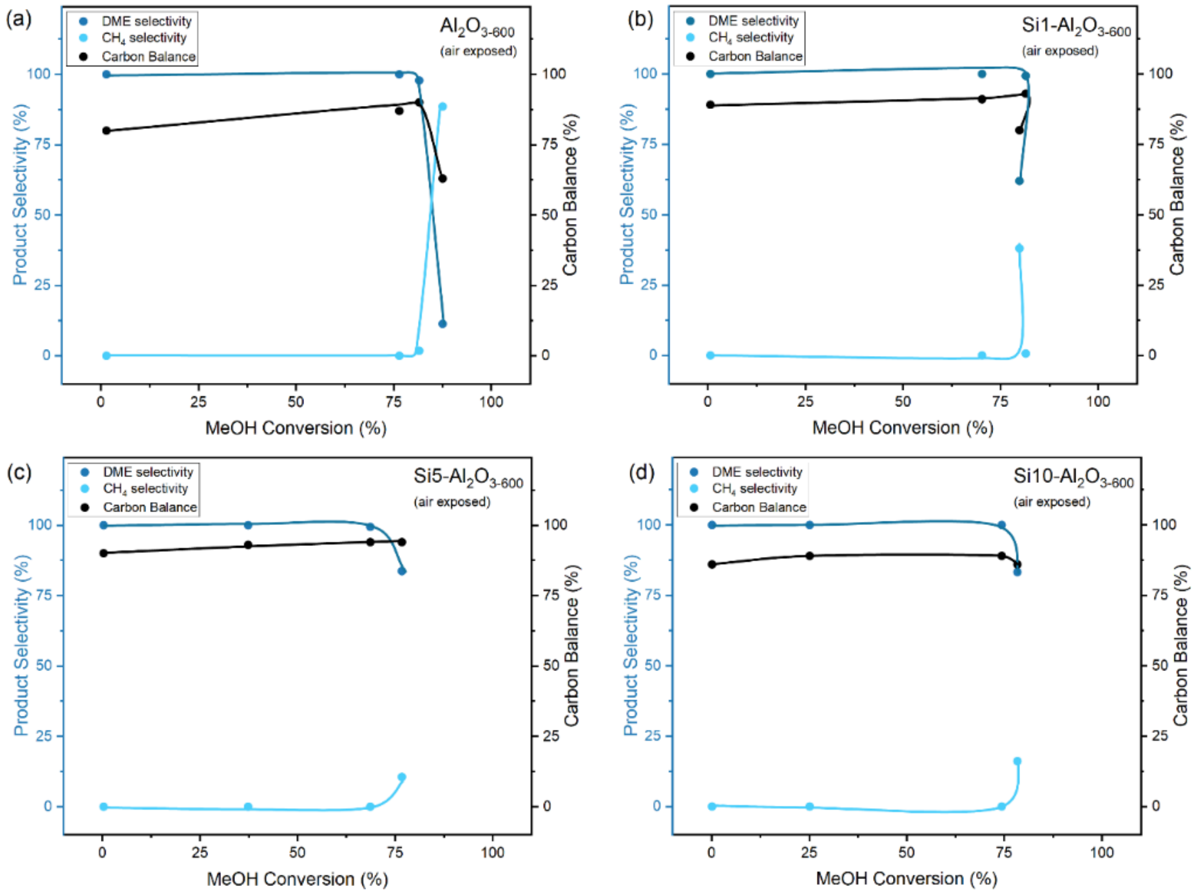

Figure S9. Selectivity to DME, and methane and carbon balance as a function of methanol conversion for (a) Al_2O_{3-600}, (b) Si1-Al_2O_{3-600}, (c) Si5-Al_2O_{3-600}, and (d) Si10-Al_2O_{3-600}.

Catalysts were loaded in air. The trendlines were added to guide the eye.

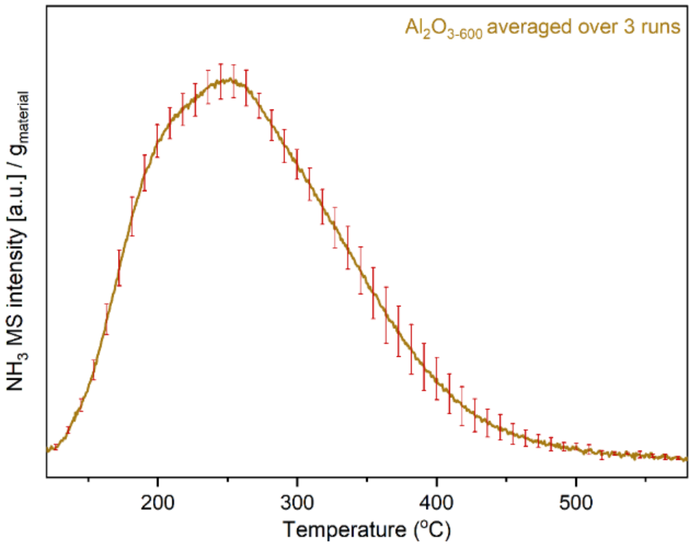

Figure S10. Average of 3 independent NH_3-TPD runs of Al_2O_{3-600} showing experimental reproducibility.

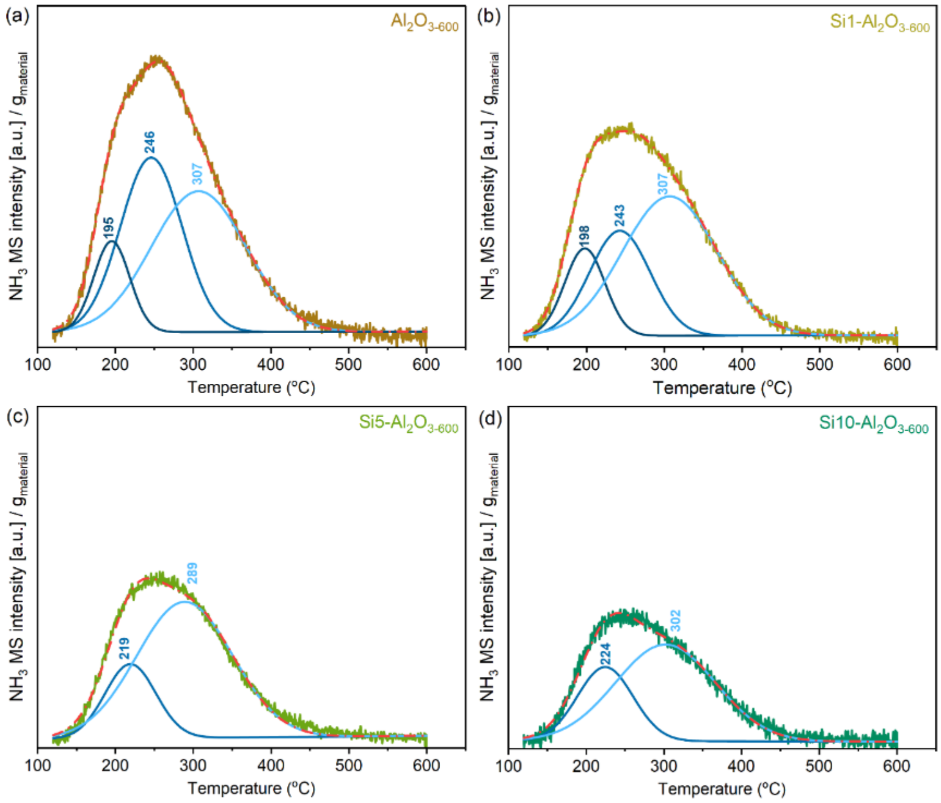

Figure S11. The deconvolution of the NH_3-TPD peaks of (a) Al_2O_{3-600}, (b) Si1-Al_2O_{3-600}, (c) Si5-Al_2O_{3-600}, (d) Si10-Al_2O_{3-600}.

The fitted curve to the cumulative data is in red dashed lines, with the individual fitted components in blue solid lines.

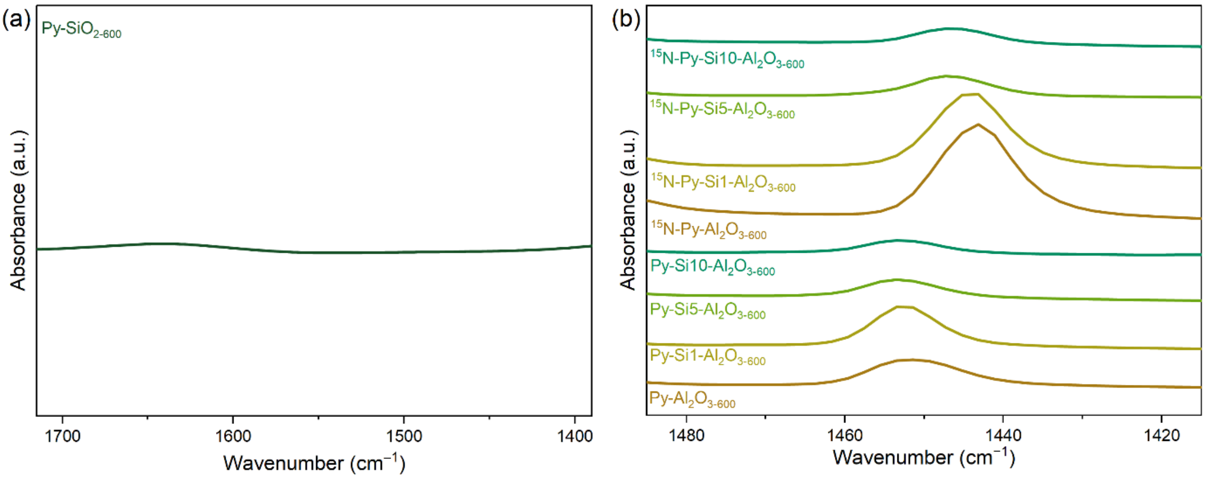

Figure S12. Py-FTIR spectra of (a) SiO_{2-600} in the 1710-1390 cm^{-1} range, and (b) ^{15}N-Py and Py-FTIR spectra of Al_2O_{3-600} and Si1-, Si5, and Si10-Al_2O_{3-600} in the 1470-1430 cm^{-1} range.

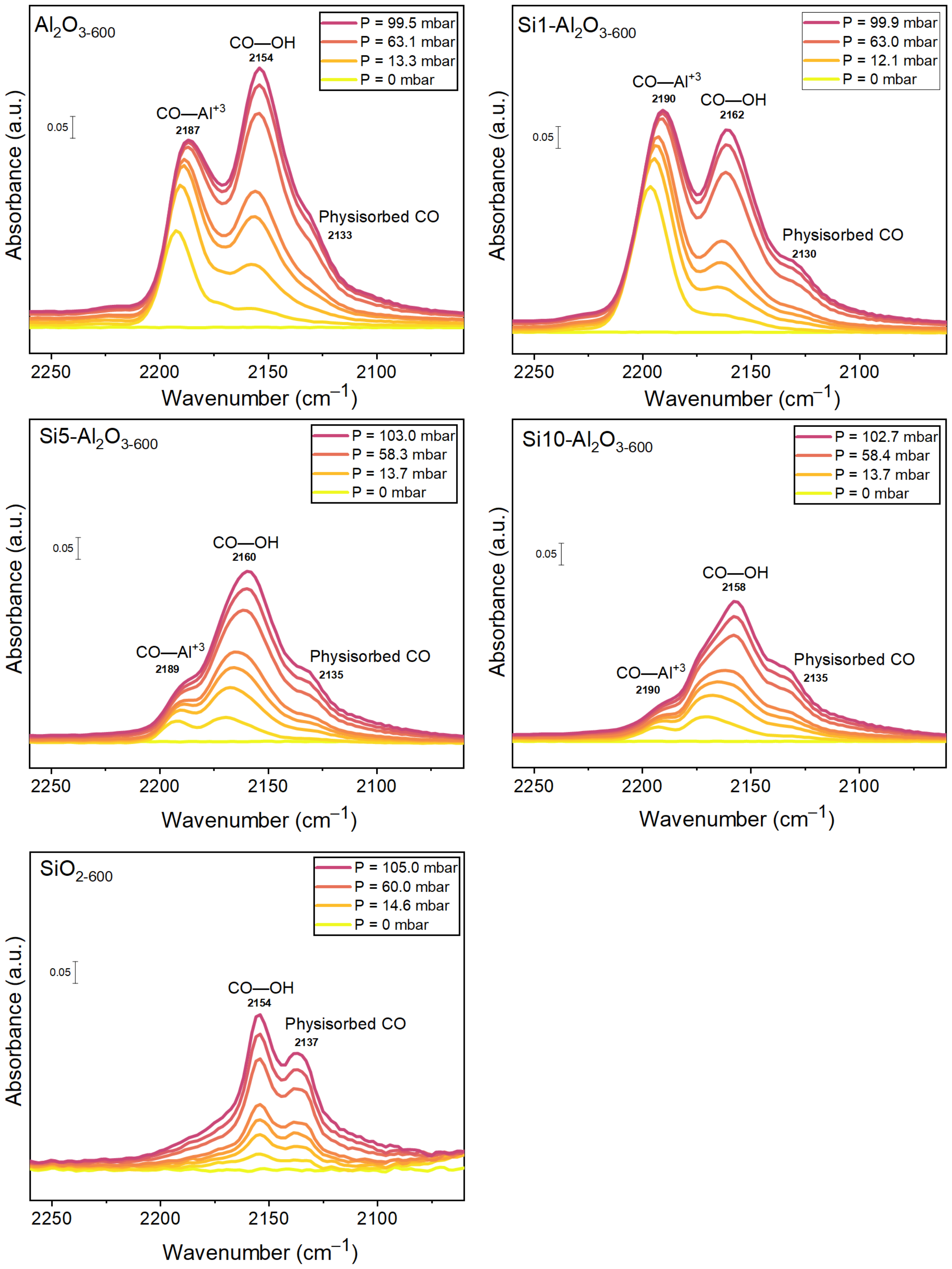

Figure S13. CO-DRIFTS profiles of Al_2O_{3-600}, Si1-, Si5, Si10-Al_2O_{3-600}, and SiO_{2-600} with increasing CO pressure.

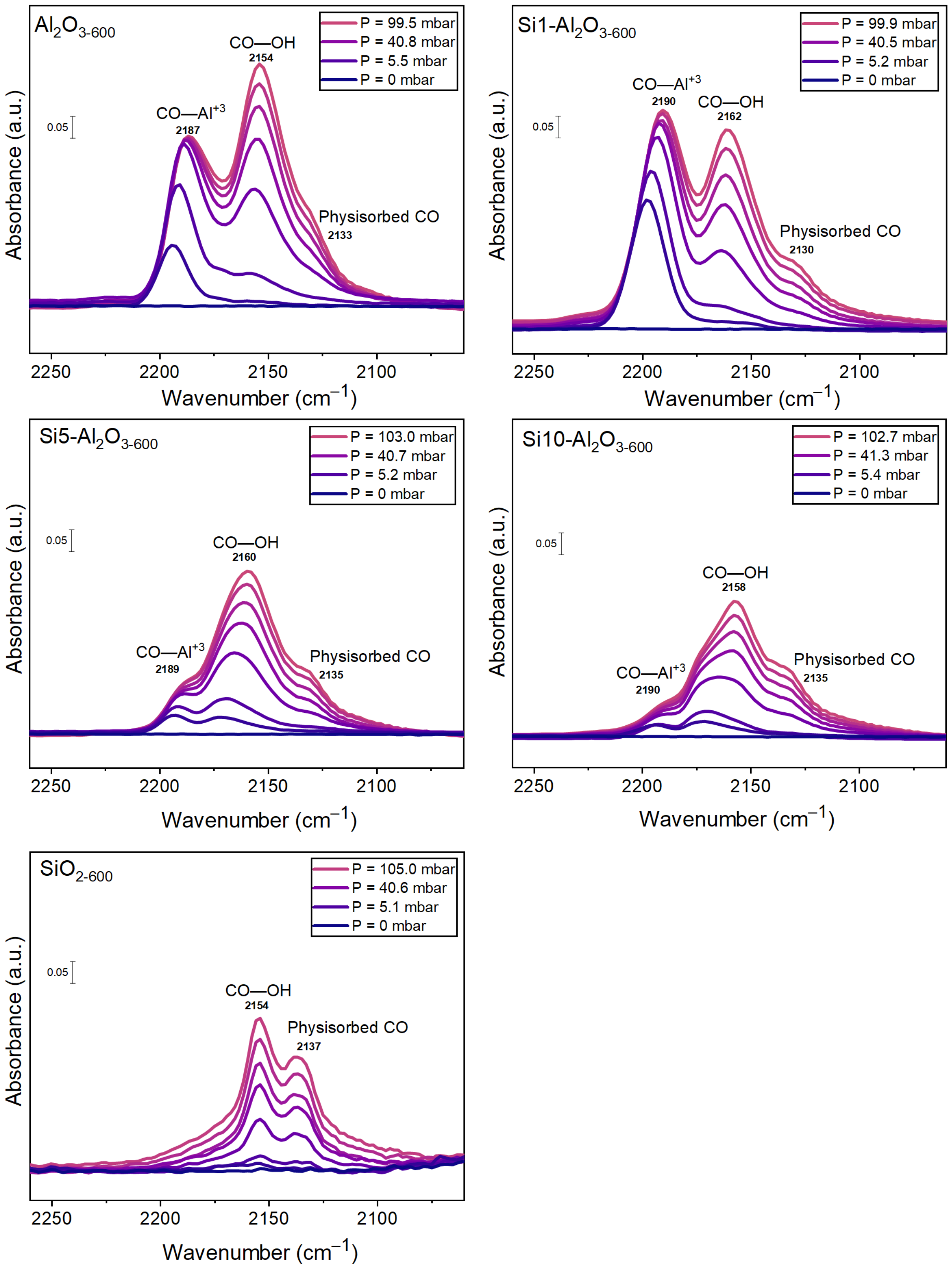

Figure S14. CO-DRIFTS profiles of Al_2O_{3-600}, Si1, Si5, Si10-Al_2O_{3-600}, and SiO_{2-600} for decreasing CO pressure.

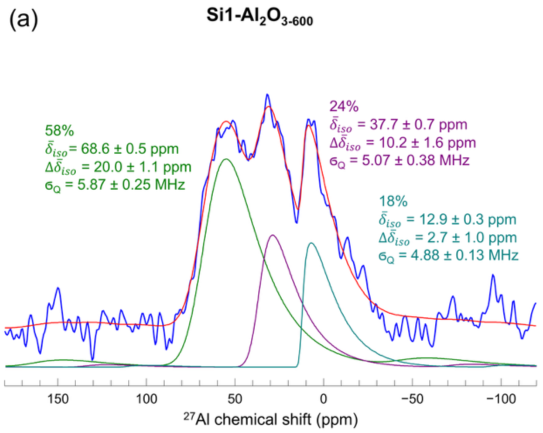

Figure S15. Simulations of $^{27}Al\{^{29}Si\}$ SR4_1^2 D-HMQC spectra of (a) Si1-Al_2O_{3-600} and (b) Si10-Al_2O_{3-600} using 6.0 ms for recoupling time.

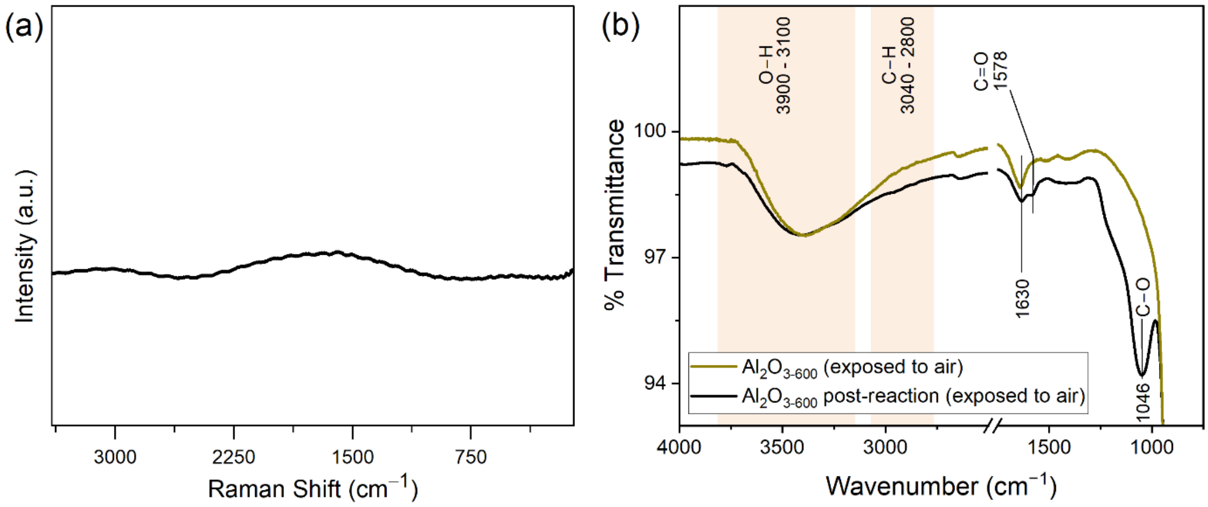

Figure S16. (a) Raman spectrum of post-reaction $Al_2O_{3\text{–}600}$ exposed to air and (b) attenuated total reflectance IR spectra of the fresh and post-reaction $Al_2O_{3\text{–}600}$, exposed to air.

Supporting Tables

Table S1. BET surface area, pore volume, and pore diameter of Al_2O_{3-600}, Si1,5,10-Al_2O_{3-600}, and SiO_{2-600} ambient air-exposed materials.

Material	BET surface area (m^2 g^{-1})	Pore volume (cm^3 g^{-1})	Pore diameter (nm)	Si weight fraction (%)	Si surface density (Si nm^{-2})
Al_2O_{3-600}	150	0.4	9.4		
Si1-Al_2O_{3-600}	154	0.4	9.4	1.1	1.5
Si5-Al_2O_{3-600}	169	0.4	8.8	5.9	8.3
Si10-Al_2O_{3-600}	168	0.4	8.1	9.9	13.7
SiO_{2-600}	265	1.5	22.1		

Table S2. Fitting parameters of the ^{29}Si DNP SENS data.

Material	Site	$\bar{\delta}_{iso}$ (ppm)	$\Delta\delta_{iso}$ (ppm)	Area (%)
Si1-Al_2O_{3-600}	1	–82.6	7.6	70.4
	2	–89.1	5.3	18.8
	3	–92.8	8.2	10.8
Si5-Al_2O_{3-600}	1	–85.9	8.4	13.1
	2	–92.4	7.3	22.4
	3	–99.9	12.3	64.5
Si10-Al_2O_{3-600}	1	–86.2	3.2	2.4
	2	–93.1	5.6	17.1
	3	–101.6	12.7	80.6

Table S3. Results of the catalytic tests.

Entry	Catalyst	T (°C)	X_{MeOH} (%)	S_{DME} (%)	S_{CH4} (%)	Carbon Balance (%)	STY_{DME} (g_{DME} (g_{cat} h)$^{-1}$)
1	Al_2O_{3-600}	150	1.35	100	0	80	0.020
		250	76.4	100	0	87	1.25
		350	81.5	97.8	1.8	90	1.36
		450	87.5	11.4	88.6	63	0.119
2*	Al_2O_{3-600}	150	1.66	100	0	78	0.027
		250	76.8	100	0	83	1.32
		350	81.6	97.8	2.2	86	1.42
		450	89.1	13.9	86.1	61	0.157
3	Si1-Al_2O_{3-600}	150	0.60	100	0	89	0.010
		250	70.2	100	0	91	1.24
		350	81.4	99.3	0.7	93	1.46
		450	79.8	62.0	38.0	80	0.772
4	Si5-Al_2O_{3-600}	150	0.34	100	0	90	0.006
		250	37.3	100	0	93	0.698
		350	68.6	99.4	0	94	1.29
		450	76.7	83.7	10.6	94	1.21
5	Si10-Al_2O_{3-600}	150	0.13	100	0	86	0.002
		250	25.1	100	0	89	0.437
		350	74.3	100	0	89	1.29
		450	78.3	83.3	16.1	86	1.10
6	SiO_{2-600}	150	0	0	0	86	0
		250	0.04	100	0	95	0.001
		350	0.50	100	0	99	0.009
		450	4.4	100	0	95	0.079

* The catalytic test for Al_2O_{3-600} was performed without exposing the dehydroxylated material to ambient air. For the other tests, materials were loaded into reactors in air.

Table S4. The amount of LAS calculated from the Py-FTIR spectra and the total number of acidic sites calculated from ammonia TPD profiles.

Material	LAS and BAS amount (μmol g^{-1})	LAS and BAS amount (molecules nm^{-2})	LAS amount (μmol g^{-1})	LAS amount (molecules nm^{-2})
Al_2O_{3-600}	316	1.26	130	0.52
Si1-Al_2O_{3-600}	259	1.01	135	0.53
Si5-Al_2O_{3-600}	202	0.72	68	0.24
Si10-Al_2O_{3-600}	157	0.56	51	0.18

Table S5. Parameters obtained by fitting the ^{15}N DNP SENS data.

Material	Site	$\bar{\delta}_{iso}$ (ppm)	$\Delta\delta_{iso}$ (ppm)	Area (%)
Al_2O_{3-600}	Py-$L_{(1)}$	278	12.5	10
	Py-$L_{(2)}$	265	19.7	62
	Py-$L_{(3)}$	239	12.0	27
	$PyH^+_{(1)}$	–	–	–
	$PyH^+_{(2)}$	–	–	–
Si1-Al_2O_{3-600}	Py-$L_{(1)}$	275	11.6	13
	Py-$L_{(2)}$	262	19.4	57
	Py-$L_{(3)}$	237	12.3	27
	$PyH^+_{(1)}$	211	14.9	3
	$PyH^+_{(2)}$	–	–	–
Si5-Al_2O_{3-600}	Py-$L_{(1)}$	–	–	–
	Py-$L_{(2)}$	260	19.0	25
	Py-$L_{(3)}$	235	15.6	20
	$PyH^+_{(1)}$	207	16.1	24
	$PyH^+_{(2)}$	200	9.4	31
Si10-Al_2O_{3-600}	Py-$L_{(1)}$	–	–	–
	Py-$L_{(2)}$	260	18.6	34
	Py-$L_{(3)}$	235	15.2	25
	$PyH^+_{(1)}$	205	13.6	22
	$PyH^+_{(2)}$	197	18.6	18

References

(1) Copéret, C. C.; Comas-Vives, A.; Conley, M. P.; Estes, D. P.; Fedorov, A.; Mougel, V.; Nagae, H.; Núñez-Zarur, F. N.; Zhizhko, P. A. Surface Organometallic and Coordination Chemistry toward Single-Site Heterogeneous Catalysts: Strategies, Methods, Structures, and Activities. *Chem. Rev.* **2016**, *116*, 323–421.

(2) Zholobenko, V.; Freitas, C.; Jendrlin, M.; Bazin, P.; Travert, A.; Thibault-Starzyk, F. Probing the Acid Sites of Zeolites with Pyridine: Quantitative AGIR Measurements of the Molar Absorption Coefficients. *J. Catal.* **2020**, *385*, 52–60.

(3) Tricot, G.; Trébosc, J.; Pourpoint, F.; Gauvin, R.; Delevoye, L. The D-HMQC MAS-NMR Technique: An Efficient Tool for the Editing of Through-Space Correlation Spectra Between Quadrupolar and Spin-1/2 (^{31}P, ^{29}Si, ^{1}H, ^{13}C) Nuclei. *Annu. Rep. NMR Spectrosc.* **2014**, *81*, 145–184.

(4) Massiot, D.; Fayon, F.; Capron, M.; King, I.; Le Calvé, S.; Alonso, B.; Durand, J. O.; Bujoli, B.; Gan, Z.; Hoatson, G. Modelling One- and Two-Dimensional Solid-State NMR Spectra. *Magn. Reson. Chem.* **2002**, *40*, 70–76.

(5) Le Caër, G.; Brand, R. A. General Models for the Distributions of Electric Field Gradients in Disordered Solids. *J. Phys.: Condens. Matter* **1998**, *10*, 10715–10774.
